# Fabrication of an Organic–Inorganic Hybrid Hard Coat with a Gradient Structure Controlled by Photoirradiation

**DOI:** 10.1021/acsami.3c04399

**Published:** 2023-06-05

**Authors:** Yoshiki Shirai, Ayano Sasaki, Sayako Sato, Daisuke Aoki, Koji Arimitsu

**Affiliations:** † Department of Pure and Applied Chemistry, Faculty of Science and Technology, Tokyo University of Science, 2641 Yamazaki, Noda, Chiba 278-8510, Japan; ‡ Toyota Industries Corporation, 2-1 Toyoda-cho, Kariya-shi, Aichi 448-8671, Japan

**Keywords:** hard coating, gradient structure, photopolymerization, organic−inorganic hybrid, sol−gel reaction, photobase generator, photoradical initiator, polycarbonate

## Abstract

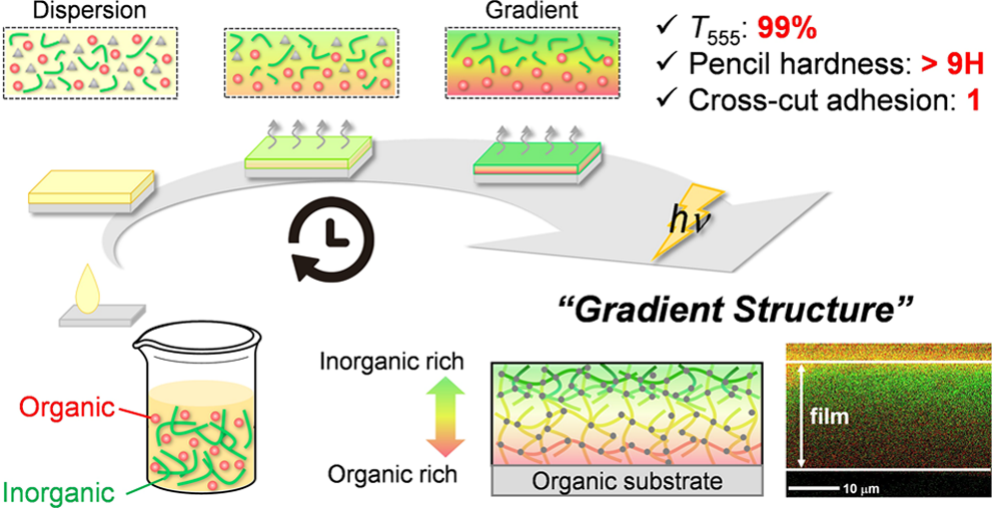

Organic–inorganic materials have attracted attention because of the advantages of both organic and inorganic resins. Among their disadvantages, hard coating films made of organic–inorganic mixtures of resins have opacity and interface peeling problems because of organic–inorganic phase separation and surface segregation of inorganic resins. Although an organic–inorganic gradient-structured material comprising an inorganic-rich domain at the air interface and an organic-rich domain at the organic substrate has the potential to solve these problems, the fabrication of a gradient-structured material has not yet been achieved. Here, we describe the fabrication of an organic–inorganic gradient film by impeding the movement of organic and inorganic resins through radical photopolymerization of organic and inorganic oligomers. Moreover, we successfully enhanced gouge hardness by cross-linking with photobase-catalyzed sol–gel reactions of inorganic resins at the air interface. As a result, the organic–inorganic gradient coating contributed excellent gouge hardness (pencil hardness >9H), adhesion to an organic substrate such as polycarbonate, and transparency (visible light transmittance >99%T). In addition, we demonstrated that the formation of organic–inorganic gradient structures is dominated by the surface free energy and viscosity of each resin. Achieving a gradient structure required a significant difference in surface free energy (>20 mJ/m^2^) and high mixture viscosity (>65 mPa·s).

## Introduction

Gradient structures are often found in nature, for example, in bamboo, bones, joints, shells, and teeth.^1,2^ These gradient structures confer high performance attributes such as elasticity, flexibility, hardness, lightness, mechanical strength, and toughness because of the gradual transitions between the different materials interfacing at a microscale level. Among them, clam hinges and coconut crab pincers achieve both hardness and flexibility by forming a gradual transition between stiff calcium carbonate calcification and soft proteins.^3,4^ These important functional attributes, acquired by the gradual transition at the heterogeneous interface between organic and inorganic matter in nature, inspired new platforms for achieving different physical properties on each side of the interface.^5,6^

However, the fabrication of artificial organic–inorganic gradient-structured materials is exceptionally challenging because of their thermodynamic instability. Specifically, gradient-structured states exist at the boundary of binodal or spinodal curves, implying an intermediate state between compatibility and incompatibility. Importantly, a gradient structure is fundamentally different from conventional organic–inorganic nanocomposites because it exists between compatible and incompatible states. While the organic–inorganic materials usually form incompatible states such as sea-island structures,^7−10^ the incorporation of additives such as fillers and coupling agents, as well as the polymerization of monomers bearing organic and inorganic groups, can form compatible states such as dispersion structures. These methods prevent resins from separating into sea-island structures.^11−13^ In essence, organic–inorganic materials are very stable for dispersion and sea-island structures. Therefore, forming structural materials with an organic–inorganic gradient requires a driving force for the transition between incompatibility and compatibility.

Coated films can potentially exploit an organic–inorganic gradient structure because of the requirements for different physical properties on both sides. In addition, coated films, owing to their vast air interface, are significantly influenced by surface free energy, sometimes leading to surface segregation phenomena. Fundamentally, this surface segregation state lifts inorganic resins to the surface using surface free energy as the driving force, which is distinct from the mechanisms forming sea-island and dispersion structures. Surfaces with low free energy groups such as fluorinated polyhedral oligomeric silsesquioxane (POSS), polyurethane copolymer bearing poly(dimethylsiloxane) (PDMS), and poly(perfluoroalkylethyl methacrylate) induce segregated structures. These examples suggest surface segregation of inorganic resins by XPS or contact angle measurements.^14−17^

However, the compatibility of organic and inorganic resins in gradient-like structures by preventing the change from dispersion to segregation structures has not yet been achieved. Furthermore, the segregation process of inorganic resins has remained elusive, making gradient structure design difficult. Therefore, we hypothesized that an organic–inorganic gradient structure could be produced by preventing the segregating process by using rapid photopolymerization.

In the present report, we describe simple methods for producing organic–inorganic gradient-structured films with cross-section SEM-EDX measurements, in contrast with sea-island, dispersion, and segregation structures, elucidating the segregation process, driving force, and parameters for producing a gradient structure. We added solvent as a compatibilizer and used monomers or oligomers to temporarily mix organic and inorganic resins. In addition, we prevented the process of segregating the coated organic monomers and inorganic oligomers bearing photopolymerizable groups, allowing gradient-structured films. Furthermore, we describe the simple methods to realize the organic–inorganic gradient structure and the proposed important parameters, such as surface free energy (γ) and viscosity (η), for gradient structure formation.

## Results and Discussion

**Figure 1:**
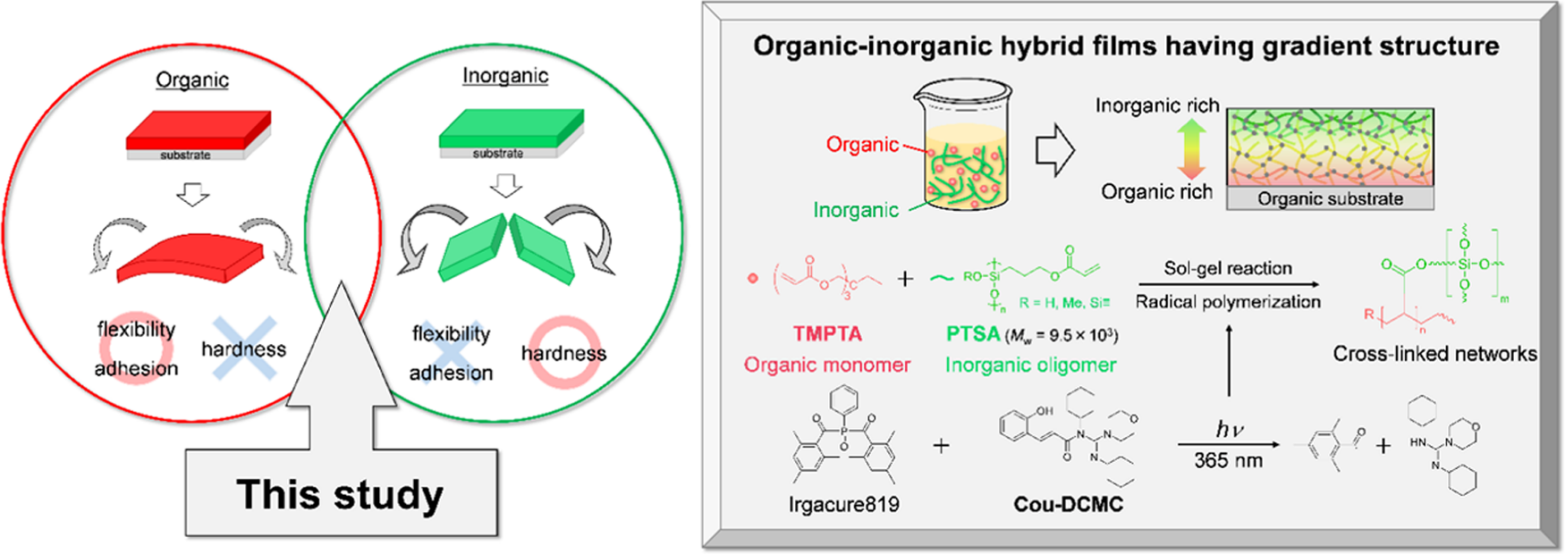
Fabrication of organic–inorganic hybrid films having gradient structure by radical-anionic UV curing.

Our general strategy for producing the organic–inorganic gradient structure is based on inducing surface segregation of organic and inorganic resins and subsequently stopping the segregating process through radical-anionic UV curing (Figure 1). To facilitate surface segregation, we selected combinations of organic monomers and inorganic oligomers with a significant difference in surface free energy. Here, radical photopolymerization allows the segregating process to be stopped quickly, whereas anionic photopolymerization by photobase generators (PBGs) simultaneously promotes the sol–gel reaction in inorganic oligomers. In the following discussion, we explain the production of organic–inorganic gradient coating films using **TMPTA** and **PTSA** as representative organic and inorganic resins. **TMPTA** is a trifunctional acrylate monomer, and **PTSA** is a silicone oligomer bearing acryloyl groups. We observed all gradient structures using SEM-EDX images of film cross-sections (Table S7).

**Figure 2:**
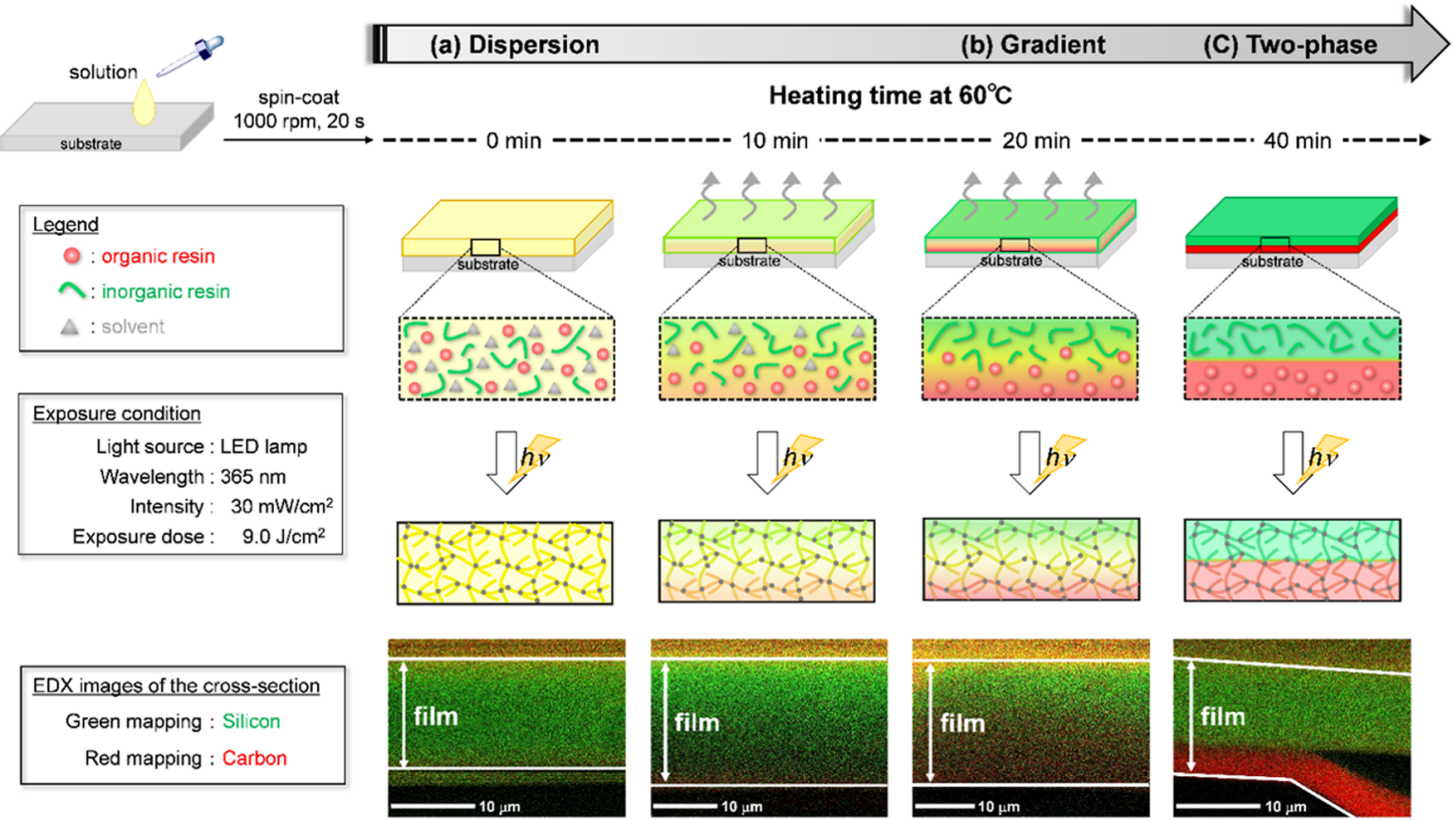
Organic and inorganic resins flow and production of the gradient structure by UV curing. (a) Cured films having a dispersion structure as a result of no heating (heating for 0 min), (b) gradient structure by heating for 20 min, and (c) two-phase separation structure by overheating (heating for 40 min). EDX maps showing the distribution of silicon (Si) and carbon (C).

First, an organic monomer (**TMPTA**), an inorganic oligomer (**PTSA**), and a photoradical initiator (Irgacure 819) were dissolved in carbon tetrachloride (CCl_4_). After the solution was spin-coated on a polycarbonate substrate (1000 rpm, 20 s), the films were heated at 60 °C to remove CCl_4_. In this process, evaporating the solvent by heating plays a critical role in the slow surface segregation due to increasing mixture viscosity, and UV curing (30 mW/cm^2^, 9 J/cm^2^) can appropriately stop the segregation process (Figure 2). Indeed, the spin-coated films without solvent removal by heating resulted in dispersion structures because resins were not induced due to the movement of evaporating solvent and the inadequate effect of surface free energy (Figure 2a). In contrast, the organic–inorganic gradient coating is obtained under conditions (heating for 20 min) intermediate between those for dispersed and segregated structures (Figure 2b). However, overly accelerated segregation (heating for 40 min) led to a completely vertically separated bilayer (Figure 2c). Consequently, this process can solve the interface peeling problem of complete two-phase separation between the organic and inorganic layers because of the gradual transition between the organic–inorganic interface. After heating to transform the organic and inorganic resin mixture to a gradient structure, UV irradiation at 365 nm allows curing by radical and anionic polymerization. Figure S4 shows the acrylate olefins of **TMPTA** and **PTSA** depending on the exposure dose on the FT-IR spectra for the spin-coated films. The consumption of the olefine peak by UV irradiation indicates that the radical polymerization was almost complete after 3 J/cm^2^ irradiation; hence, we unified exposure as 9 J/cm^2^ only in the radical polymerization processes.

**Figure 3:**
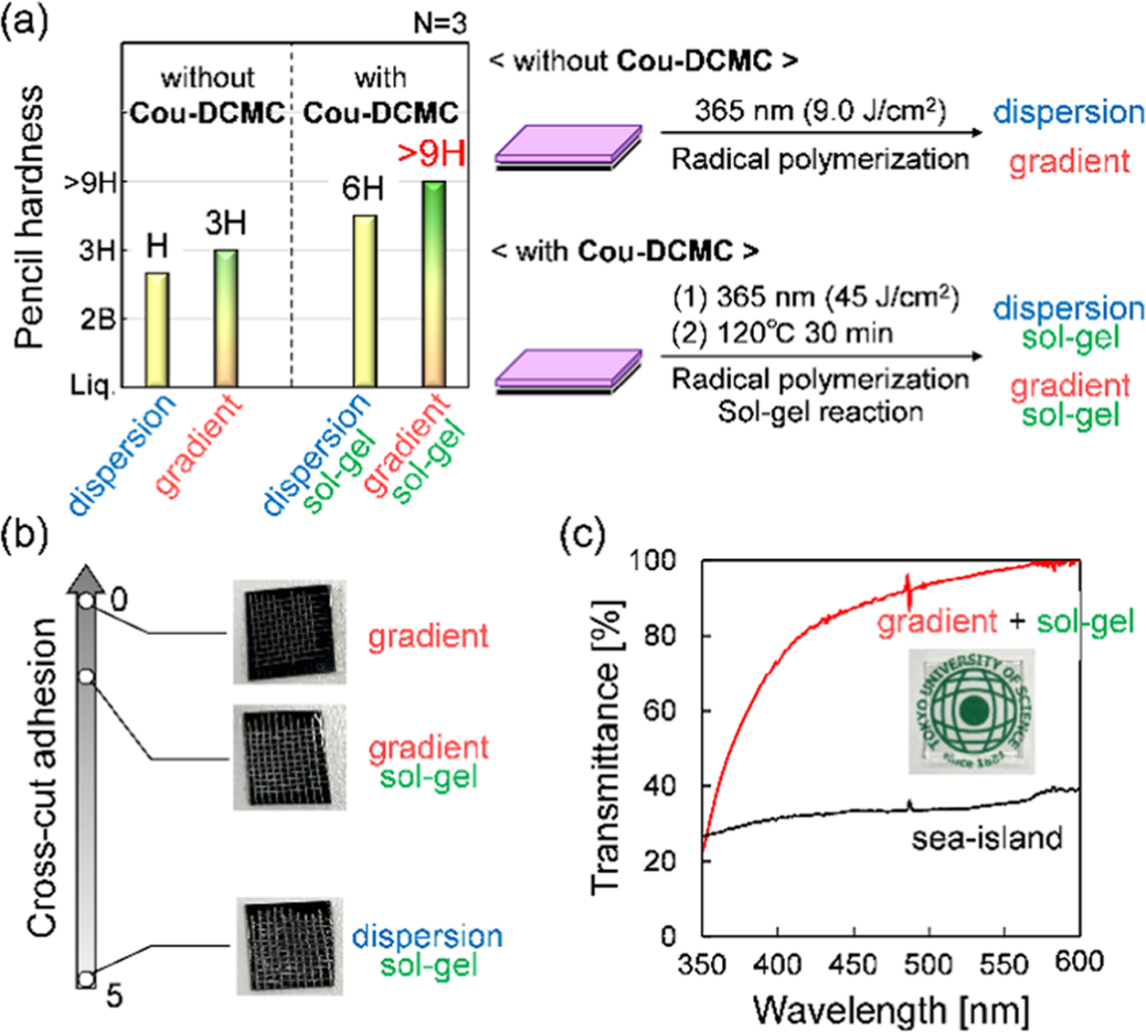
(a) Pencil (gouge) hardness of each film, (b) the cross-cut adhesion for each film, and (c) transmittance of gradient and sea-island structures by UV–vis spectra.

Figure 3a shows the pencil (gouge) hardness of **TMPTA**–**PTSA** films forming dispersion and gradient structures with or without sol–gel reactions. The pencil hardness tests were conducted, as described below. We fabricated three or more films under the same conditions, and subsequently, each film was scratched in three places using a pencil hardness tester, with the pencil held firmly against the film at a 45° angle in a 10 mm/s stroke. When every part of the film was gouge-free, the hardness of the pencil (graphite hardness) was increased by one step, and the film was tested in the same manner. The test was conducted until the scratched film was gouged, and the preceding hardness was defined as the “pencil hardness” of the film. In other words, we defined “pencil hardness” as the minimum gouge-free hardness value of the pencils. The cured films provided H, 3H, 6H, and >9H pencil (gouge) hardness properties. The gradient-structured films had excellent gouge hardness (pencil hardness 3H). In contrast, the dispersion structured films show low gouge hardness (pencil hardness H). Furthermore, the irradiated (45 J/cm^2^) and heated (120 °C, 30 min) films, including PBG (**Cou-DCMC**^18^), had enhanced gouge hardness (pencil hardness >9H) as a result of cross-linking using photobase-catalyzed sol–gel reactions of inorganic oligomer (**PTSA**) at the air interface. At the time, the adhesion of films made with these substances was measured in a cross-cut adhesion test. The gradient films had excellent adhesion to polycarbonate substrates (cross-cut adhesion test 0), unlike the dispersion film (Figure 3b). It is noteworthy that both pencil (gouge) hardness and adhesion were affected by film thickness. Thicker films resulted in higher gouge hardness with less influence from the soft polycarbonate substrate. However, although thicker films improved gouge hardness, they compromised adhesion to substrates due to the loss of film suppleness, except for the gradient structure. These results indicate that the gradient structure effectively confers gouge hardness and adhesiveness.

In addition, organic–inorganic gradient and uniform dispersion films were colorless and had optical transparency (visible light, 555 nm transmittance >99%T) compared with sea-island structured films, which had nonuniform dispersion and were opaque (Figures 3c and S5). Thus, the organic–inorganic gradient-structured films achieved unique properties, including the advantages of traditional organic–inorganic hybrid films, such as dispersed and multilayer structures, without their disadvantages.^19−24^

### Solvent Evaporation Dependency of the Gradient Structure

Next, we focused on important parameters for forming the organic–inorganic gradient structure: the viscosity of each resin for controlling the separation rate and surface free energy for stability at the air interface.^25−28^ Because solvent removal increases the mixture viscosity and decreases the compatibility of organic-to-inorganic resins, we studied their dependency on solvent evaporation. We investigated heating as a method for solvent removal, examining evaporation time and rate for **TMPTA**–**PTSA** films cured by radical photopolymerization after spin-coating. To simplify the system, we conducted the following experiment without PBG.

**Figure 4:**
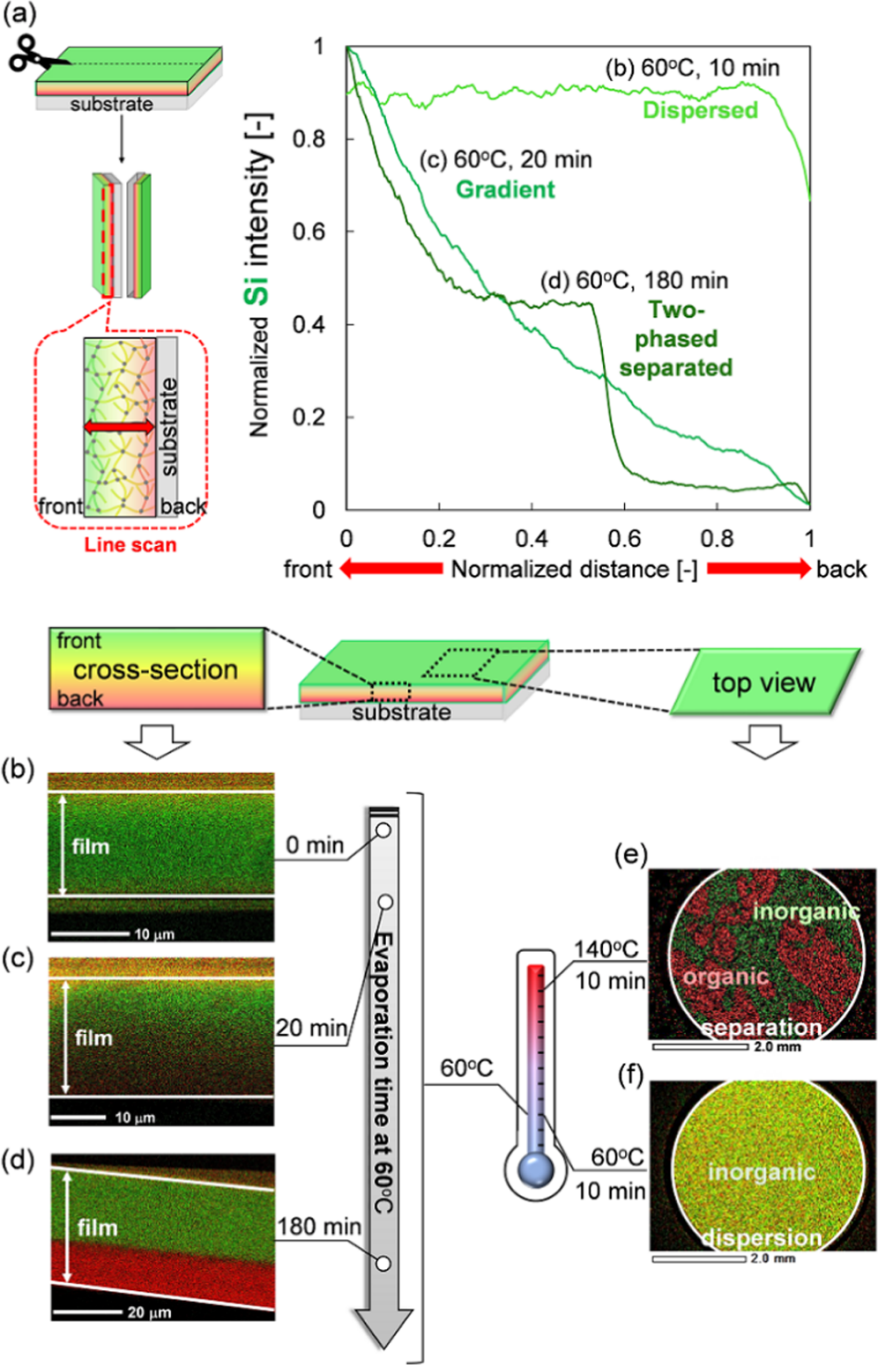
Dependence of silicon (Si) and carbon (C) distribution on solvent evaporation rate and time measured using SEM-EDX. (a) Normalized line scan of silicon density in cross-sections of cured films from the upper to lower layers, EDX images of cross-sections of films cured with (b) no heating, (c) heating for 20 min, and (d) heating for 180 min at the time of line scan, and (e, f) EDX images (top view) of surface-cured films heated at (e) 140 °C and (f) 60 °C.

We describe solvent evaporation time dependency using several cured films fabricated using various heating times (0–180 min at 60 °C). Figure 4a shows a normalized line scan of silicon intensity in a cross-section of cured films from the upper to lower layer using SEM-EDX measurements. The line scan indicates that the silicon density of the upper to the lower layer was constant with no heating, whereas it was decreased by heating. Moreover, the silicon line scan of the overheated film showed a step-like curve. The resultant film structure changed from a dispersion to a gradient to a two-phase-separated structure according to the heating time. Therefore, the gradient structure was characterized by a continuous decrease in the distribution of silicon atoms in EDX intensity from the upper to the lower layer of the films. In addition, we investigated EDX images of cross-section-cured films at the time of the line scan (Figure 4b–d). The EDX images revealed that the film cured without heating formed a dispersed structure (Figure 4b), and the film cured by heating for 20 min at 60 °C formed a gradient structure (Figure 4c). Heating for 180 min at 60 °C led to a two-phase separated structure (Figure 4d). The EDX results were consistent with the line scan results. These results indicated that a given solvent evaporation time was required for the formation of the gradient structure film because the compatibilizing solvent removal induced surface segregation of inorganic resins with low surface free energy. Thinner films require shorter solvent evaporation times for segregation due to the influence of the air interface: a phenomenon detailed in the following section. We demonstrated that the gradient structure was formed by preventing the organic and inorganic resins from segregating using UV curing.

Furthermore, we investigated the dependency of the solvent evaporation rate using films cured at two different temperatures for 10 min. Figure 4e,f shows EDX images (top view) of the surface of cured films after heating at 140 or 60 °C. The surface of the film cured by heating at 60 °C was homogeneous. However, the surface of the film cured by heating at 140 °C had a sea-island structure because of the high rate of solvent evaporation at this temperature (Figure 4e,f). The high solvent evaporation rate does not provide sufficient time to stabilize the surface free energy. In addition, a rapid increase in coated resin viscosity by rapid solvent evaporation makes it difficult to control the movement of resins.

Thus, the higher temperature induced the formation of a sea-island structure and not a homogeneous structure. Incidentally, the cross-section of the film in Figure 4f had a gradient structure, corresponding to the line scan result. Therefore, an appropriate heating time is required to produce organic–inorganic resins with a gradient structure. Furthermore, a low solvent evaporation rate can suppress sea-island formation. We found that the solvent removal rate by evaporation and the resulting viscosity change were critical for forming a gradient structure. Moreover, a gradient structure can be formed regardless of solvent type, including methanol, ethanol, toluene, high boiling point solvent, or mixed solvent, supporting our hypothesis of two important factors (Figure S6).

### Elucidation of Parameters for the Gradient Structure

**Figure 5:**
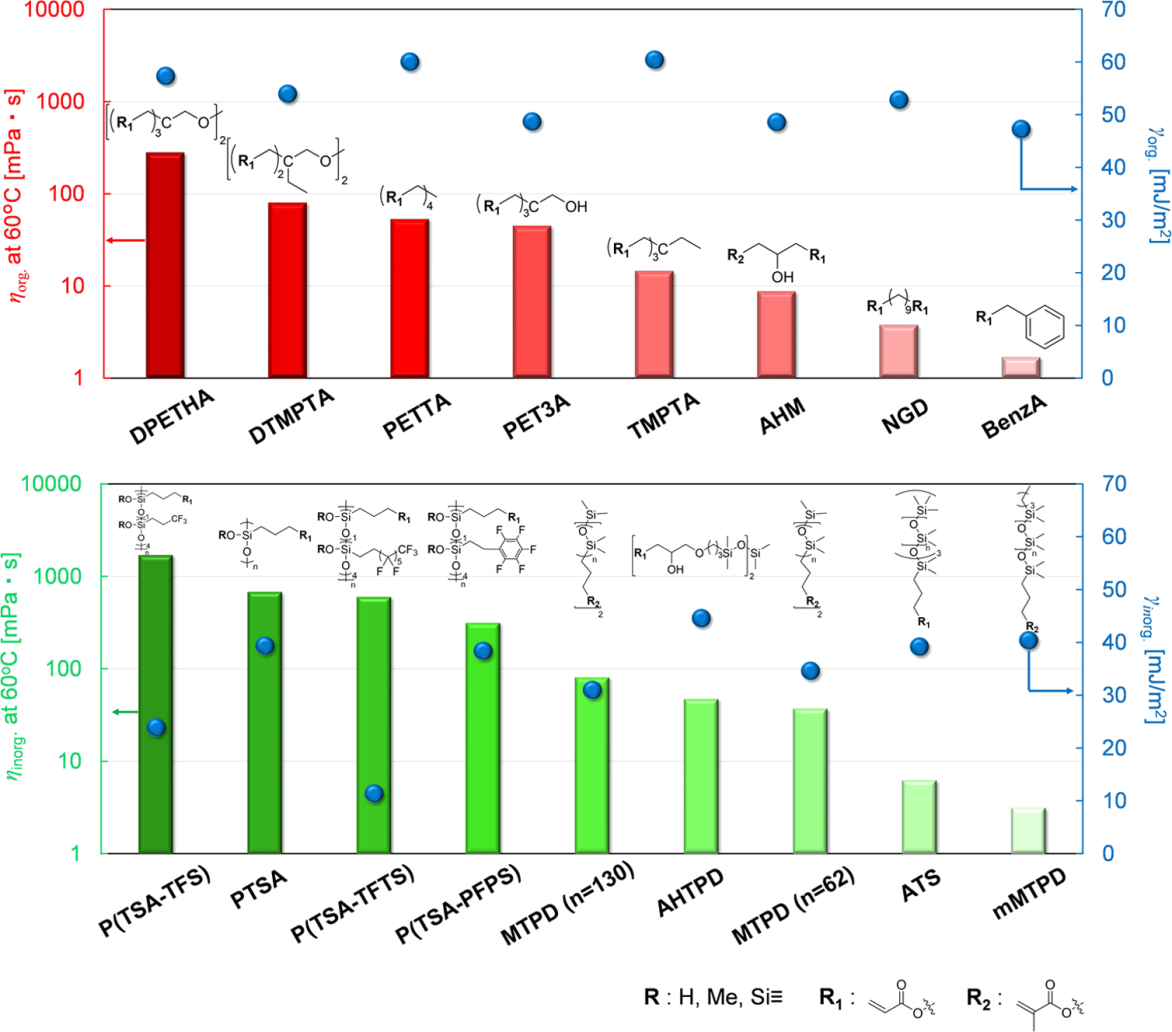
Surface free energy and the viscosity of organic and inorganic resins.

We investigated the relationship between the surface free energy (γ) and viscosity (η) of organic and inorganic resins to establish which factors control the gradient structure. Figure 5 summarizes the surface free energy and viscosity of all resins used in this work. For organic monomers, we used mono-, bi-, tetra-, or hexafunctional organic acrylic monomers (γ_org_: 47.3–60.5 mJ/m^2^, η_org_: 1.68–281.2 mPa·s). For inorganic silicone oligomers bearing acrylate or methacrylate, we used oligomers of siloxane and fluorine backbones for their low surface free energy (γ_inorg_: 11.4–44.6 mJ/m^2^, η_inorg_: 3.14–680 mPa·s).^29,30^ The surface free energy of all resins was calculated using acid–base or Owens–Wendt theory, and the viscosity of all mixture resins was calculated using the measured viscosity value of each resin at 60 °C.^31−33^

**Figure 6:**
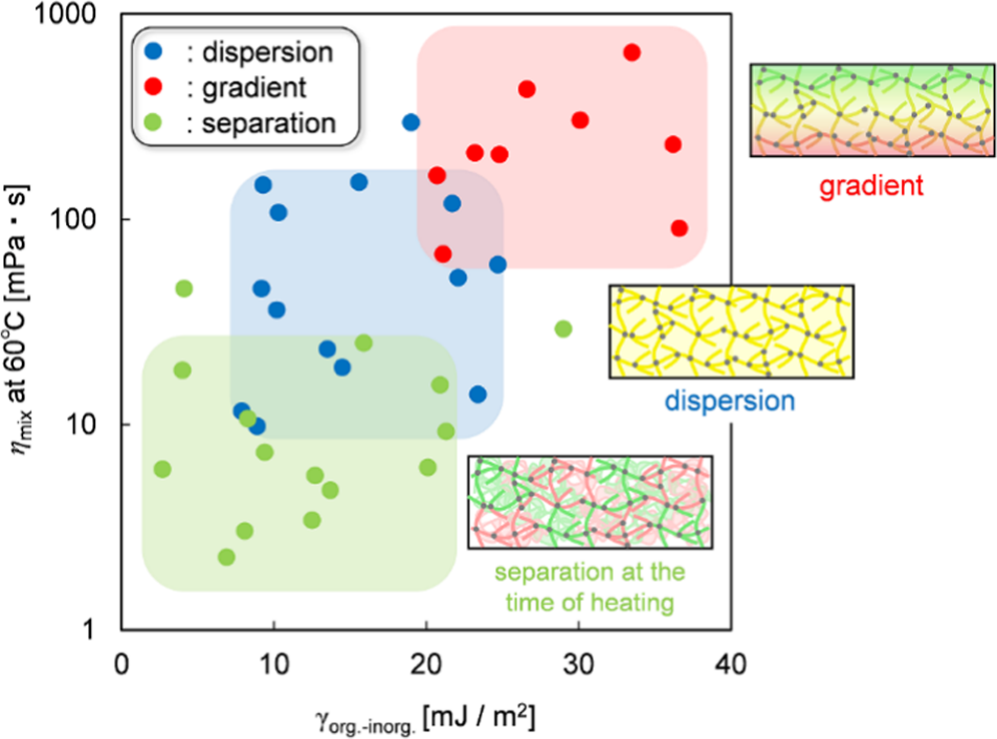
Scatterplot showing the correlation between surface free energy, mixed viscosity, and film states. The red, blue, and green plots show gradient, dispersed, and separated structures, respectively.

We presumed that the force driving the movement of the resin is surface free energy as stability at the air interface and the factor controlling the rate of movement is viscosity. Thus, we investigated organic monomers and inorganic oligomers with various surface free energies and mixture viscosities with 60 °C as the solvent evaporation temperature. Figure 6 shows the correlation between surface free energy, mixture viscosity, and film states. As the difference in surface free energy increased and the mixture viscosity increased, the resulting film structure changed from separated to dispersed to gradient. Thus, achieving the gradient structure required a substantial difference in surface free energy (>20 mJ/m^2^) and high mixture viscosity (>65 mPa·s). This finding indicates that the surface free energy difference between organic monomers as instability at the air interface and inorganic oligomers as stability at the air interface induced surface segregation of inorganic resins. Moreover, organic–inorganic mixture resins with low viscosity separated when heating due to solvent evaporation. Low-viscosity films are more affected by solvent evaporation than surface free energy as the driving force of resin movement due to their rapid separation for rapid solvent removal. In contrast, high-viscosity films are more affected by surface free energy due to their gradual separation. However, resins with small surface free energy differences formed a dispersed structure even with high viscosity because of the inadequate driving force.

Furthermore, we investigated the movement of coated resins that easily affect surface free energy or viscosity using a solvent-free coating (detailed in Figures S7 and S8). After standing for 30 min at room temperature, **TMPTA**–**PTSA-**Irgacure 651 (**TMPTA** solvent) films with high viscosity (η_high_: 537 mPa·s) formed a dispersed structure when the films were thick (*d*_thick_ > 55 μm) and a gradient structure when the films were thin (*d*_thin_ < 55 μm). The thickness of all films was measured using SEM images of cross-sections. This behavior is because a film thickness of ∼55 μm represents a threshold at which the balance between surface free energy and viscosity shifts. In films thicker than 55 μm, the suppression of resin movement by excessively high viscosity inhibited the effect of surface free energy, as only a small portion of resins in the entire coated film is affected by the air interface. In contrast, thin films are more influenced by surface free energy, suggesting that air interface effects are more dominant than viscosity.

In addition, the thick film with viscosity lowered by heating (η_low_: 67.7 mPa·s, *d*_thick_ > 55 μm) formed a gradient structure because the decrease in viscosity easily influenced the surface free energy. Incidentally, compatibility of almost all organic–inorganic resins used in this work was difficult without solvent, suggesting that solvent played a role in inducing movement of organic and inorganic resins and in organic-to-inorganic resin compatibility.

For the reasons detailed earlier, we showed that the formation of organic–inorganic gradient structures is dominated by the surface free energy and the viscosity of each organic and inorganic resin. The results described support this hypothesis. For example, the structural change from a dispersed to a gradient structure with heating (Figure 4) is because solvent evaporation by heating induced the surface free energy as a driving force and increased viscosity. In other words, controlling the movement of resins by increased viscosity easily forms a gradient structure. Therefore, we provide evidence that surface free energy is the driving force for resin movement, and the parameter controlling the movement is viscosity. In light of this evidence, we found that the formation of a gradient structure did not depend on solubility as the known resin compatibility parameter, whereas it did rely on compatibility when the organic–inorganic solvent was prepared (Figures S11 and S12).^34,35^

We spin-coated **TMPTA**–**PTSA** solutions onto various substrates, such as acrylic plates, CaF_2_ plates, glass plates, PE films, PET films, Si wafers, and PC plates. The resultant films cured on the PC and PE plates formed a gradient structure, whereas films cured onto the other substrates formed a dispersed structure due to the interface free energy stability between **TMPTA** and the substrates. The interface free energy between **TMPTA** and the substrate (PC and PE plates) was lower than that for **TMPTA** and the other substrates.^36^ Therefore, we demonstrated that the forces driving both the surface free energy of resins and the interface free energy between resins and substrates were important for forming a gradient structure (Figure S9).

## Conclusions

We achieved an organic–inorganic gradient structure in coating films by stopping the segregation of organic and inorganic resins by radical photopolymerization. We demonstrated that slow solvent evaporation gradually caused the segregation by the surface free energy without forming a sea-island structure and afforded the photo cross-linking at an appropriate state. Moreover, we investigated the detailed mechanism for the gradient formation for photo cross-linked model resins composed of **TMPTA**, **PTSA**, and Irgacure 819 as an organic monomer, an inorganic oligomer, and a photoradical initiator. Evaporating the solvent by heating at 60 °C for an appropriate time of 10–20 min successfully achieved the desired organic–inorganic gradient structure. In contrast, heating for 0 min or 180 min resulted in a dispersed or complete two-phase separated structure, respectively. Fabricating a gradient by solvent evaporation can be achieved with various solvents, including methanol, ethanol, toluene, other high boiling point solvents, and mixing solvents. Furthermore, we elucidated the parameters for producing a gradient structure using many organic and inorganic resin combinations. The formation of a gradient structure required both a substantial difference in surface free energy (>20 mJ/m^2^) and high mixture viscosity (>65 mPa·s) at 60 °C. In addition, the organic–inorganic gradient coating contributed excellent gouge hardness (pencil hardness >9H), adhesion to an organic substrate such as polycarbonate, and transparency (visible light transmittance almost 100%).

We successfully elucidated the segregation process, driving force, and parameters for gradient structure formation by combining abundant resins and solvents. Generalized methods may be used widely to develop desired gradient-structured coating materials, such as adhesives, optical communication components, and biomaterials. We believe this report represents the first step in advancing the development of materials that combine properties previously considered incompatible.

## Supporting Information Available

Experimental details for preparation of experimental materials, SEM-EDX measurements, surface free energy measurements, various parameters of all organic and inorganic resins, and additional figures and tables (PDF)
