# Supplementary material for: Fabrication of an Organic–Inorganic Hybrid Hard Coat with a Gradient Structure Controlled by Photoirradiation
Source: ACS Appl Mater Interfaces. 2023 Jun 5;15(23):28563–9. doi: 10.1021/acsami.3c04399 (PMC10273177; doi:10.1021/acsami.3c04399)
Supplement: Supplementary file 1 — am3c04399_si_001.pdf [file am3c04399_si_001.pdf]

Supporting Information

Fabrication of an Organic–Inorganic Hybrid Hard Coat with a Gradient Structure Controlled by Photoirradiation

Yoshiki Shirai[a], Ayano Sasaki[b], Sayako Sato[b], Daisuke Aoki[a], Koji Arimitsu[a]*

[a] *Department of Pure and Applied Chemistry, Faculty of Science and Technology, Tokyo University of Science, 2641 Yamazaki, Noda, Chiba 278-8510, Japan*
[b] *Toyota Industries Corporation, 2-1 Toyoda-cho, Kariya-shi, Aichi 448-8671 Japan*

Corresponding author Email; arimitsu@rs.tus.ac.jp

Table of contents

1. Materials and Instruments
    1.1 Materials
    1.2 Instruments
2. Fabrication Conditions of Gradient Structure
3. Fabrication Conditions of Gradient Structure using Photobase-catalyzed Sol–Gel Reactions
4. Measurements Methods for This Work
5. Synthesis of Inorganic Oligomers
    5.1 PTSA
    5.2 P(TSA–TFS)
    5.3 P(TSA–TFTS)
    5.4 P(TSA–PFPS)
6. FT-IR Spectrometry for Radical Polymerization
7. Transparency of films
8. Dependence of Various Parameters for Cured Films
    8.1 Solvent dependency
    8.2 Viscosity, surface free energy and film thickness dependency of solventless films
    8.3 Substrate dependency

8.4 Standing time and film thickness dependency of solvent-free films

8.5 Film thickness dependency on surface hardness and adhesion to organic substrate

9. Various Parameters of All Organic and Inorganic Resins

9.1 Viscosity and SP value of organic reins

9.2 Viscosity and SP value of inorganic resins

9.3 Contact angles and surface free energy of organic resins

9.4 Contact angles and surface free energy of inorganic resins

10. Correlation Between Various Parameters and Film or Solution States

10.1 Correlation between solubility parameter difference, mixed viscosity in 60°C and film states

10.2 Correlation between solubility parameter difference, mixed viscosity of organic resins, inorganic resins, and solvent (CCl_4) in 25°C and membrane states

11. Mixed Viscosity

11.1 Mixed viscosity of organic resins and inorganic resins in 60°C

11.2 Calculation method of organic and inorganic mixed viscosity

12. Film or Solution State in Each Mixed Resin

13. Pencil hardness test

14. Abbreviations and Structures of the Chemical Compounds

15. References

1. ***Materials and Instruments***

1.1 Materials

1-(Acryloyloxy)-3-(methacryloyloxy)-2-propanol (**AHM**), 1,9-bis(acryloyloxy)nonane (**NGD**), 2-acetoxycinnamic acid (**2Aca-acid**), 3-(trimethoxysilyl)propyl acrylate (**TSA**), benzyl acrylate (**BenzA**), dipentaerythritol hexaacrylate (**DPETHA**), ditrimethylolpropane tetraacrylate (**DTMPTA**), *N*,*N'*-dicyclohexyl-4-morpholinecarboxamidine (**DCMC**), pentaerythritol tetraacrylate (**PETTA**), thionyl chloride, trimethoxy(1*H*,1*H*,2*H*,2*H*-tridecafluoro-*n*-octyl)silane (**TFTS**), trimethoxy(3,3,3-trifluoropropyl)silane (**TFS**), and trimethylolpropane triacrylate (**TMPTA**) were purchased from Tokyo Chemical Industry Co. (3-Acryloxy-2-hydroxypropoxypropyl) terminated polydimethylsiloxane (**AHTPD**) (average molecular weight 600–900 g/mol, viscosity 60–140 cSt), acryloxypropyl *t*-structure siloxane (**ATS**) (average molecular weight 500–900 g/mol, viscosity 10–12 cSt), methacryloxypropyl terminated polydimethylsiloxane (**MTPD**) (DMS-R18, R22 with different average molecular weight $4.5–5.5 \times 10^3$, 1.0×10^4 g/mol and viscosity 50–90, 125–250 cSt, respectively), monomethacryloxypropyl terminated polydimethylsiloxane (**mMTPD**) (average molecular weight 600–800 g/mol, viscosity 6–9 cSt), and pentafluorophenylpropyltrimethoxysilane (**PFPS**) were purchased from Gelest. Pentaerythritol triacrylate (**PET3A**) was purchased from Sigma-Aldrich. Dichloromethane (super dehydrated), hydrochloric acid, super dehydrated methanol and ultrapure water were purchased from Wako Pure Chemical Industries. 2,2-Dimethoxy-1,2-diphenylethan-1-one (**Irgacure 651**) and bis(2,4,6-trimethylbenzoyl) phenylphosphine oxide (**Irgacure 819**) were purchased from Ciba Specialty Chemicals. The abbreviations and chemical structures of all compounds used in this work are listed in Table S5.

1.2 Instruments

^{1}H, ^{13}C, and ^{29}Si NMR were recorded in THF-d_8 using a JEOL ECZ-500 spectrometer. The Fourier transform infrared spectrometry (FT-IR) spectra were measured using a Jasco FT-IR600 spectrometer. UV–vis measurements were performed using a Shimadzu MultiSpec-1500 spectrophotometer. SEM–EDX measurement were performed using a scanning electron microscope JSM-7600F (JEOL) with an energy dispersive X-ray spectrometer EX-37001 (JEOL), accelerating voltage was 19 kV. Vacuum sputter was performed using a SC-701HMC Ⅱ (Sanyu Electron Co.). Viscosity measurements were performed using an Anton Paar viscoQC 300 viscometer. The weight average molecular weight (M_w) and polydispersity index (M_w/M_n) were estimated by gel permeation chromatography using an L-2130 pump, L-2350 column oven, and L-2490 RI detector (Hitachi). The system was operated with KF-805L narrow dispersed polystyrene standards (Shodex), using THF as an eluent at 40°C. UV irradiation was performed using an Iwasaki LHPUV365 LED lamp. Spin-coating were performed using an Opticoat MS-A100 apparatus (Mikasa). Contact angles were measured using a Drop Master DMe-211 meter (Kyowa). Surface free energy of all resins were performed using contact angles. They were measured by applying 1.2 μL of ultrapure water, diiodomethane, and ethylene glycol onto films of which each resin was spin-coated on a substrate. Film hardness measurements were performed using a No. 533-M pencil hardness test (Yasuda). Film adhesive measurements were performed using a JIS-K5600-5-6 cross-cut adhesion test apparatus (Allgood).

2. *Fabrication Conditions for Gradient Structure*

Organic (0.1 g) and inorganic (0.1 g) resin mixtures were dissolved in carbon tetrachloride (0.2 g) containing Irgacure 819 (0.006 g). The solution was spin-coated on a polycarbonate substrate (1000 rpm, 20 s) and heated at 60°C for 30 min. The spin-coated films were irradiated by 365 nm light under the nitrogen gas. Figure S1 show fabrication conditions of organic–inorganic gradient structure using **TMPTA–PTSA**.

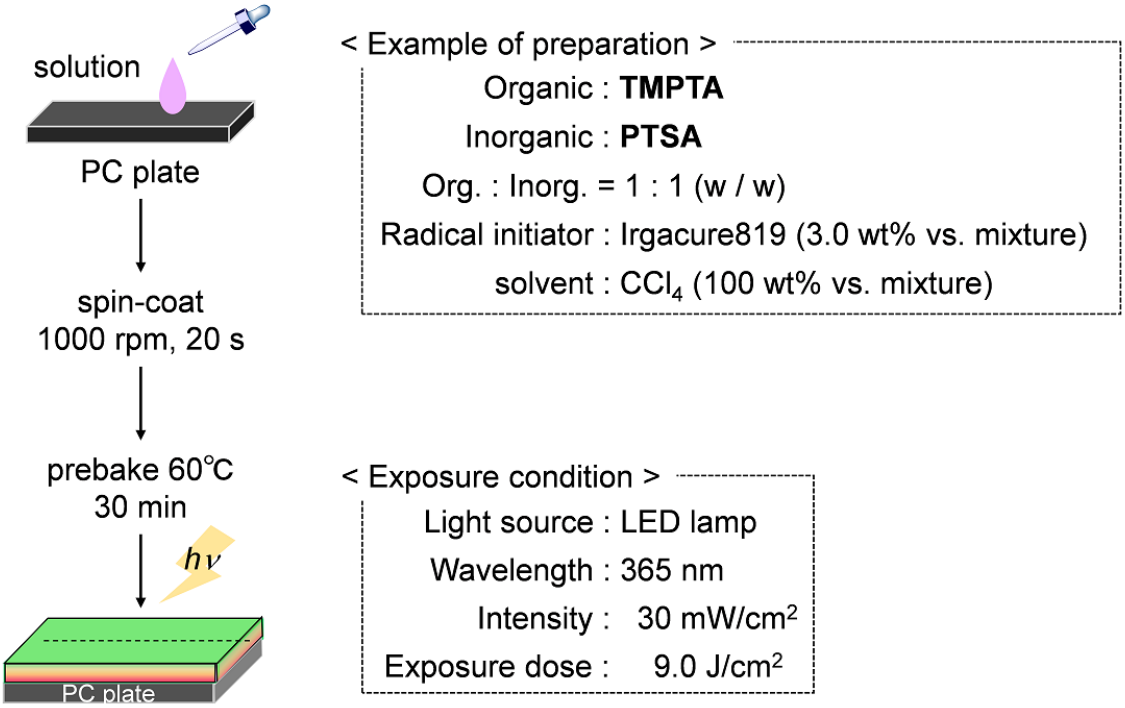

Figure S1. Fabrication conditions for gradient structure using **TMPTA–PTSA**.

3. ***Fabrication Conditions for Gradient Structure Using Photobase-catalyzed Sol–Gel Reactions***

TMPTA (0.1 g), **PTSA** (0.1 g), and **TMOS** (0.04 g) resin mixtures were dissolved in methanol (0.2 g) containing Irgacure 819 (0.006 g) and **Cou-DCMC** (0.003 g). The solution was bar-coated on a polycarbonate substrate (10 mil) and heated at 60°C for 30 min. The bar-coated cured films were heated at 120°C for 30 min. Figure S2 shows the fabrication conditions of **TMPTA–PTSA** cured films using **Cou-DCMC**-catalyzed sol–gel reactions.

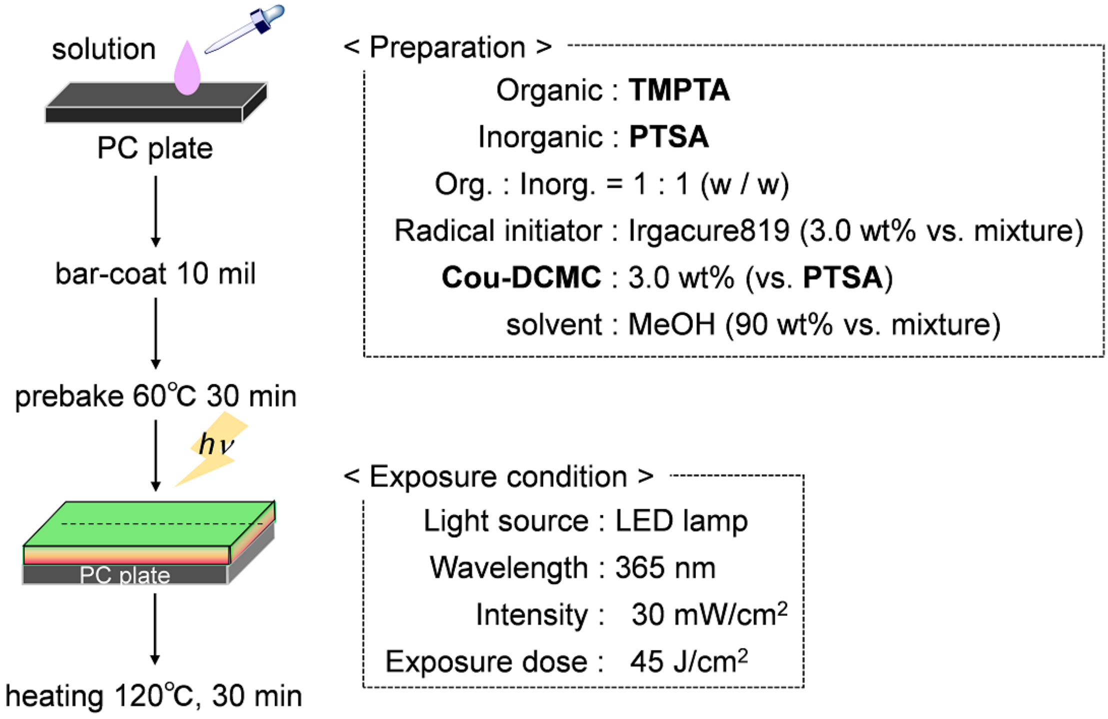

Figure S2. Fabrication conditions for **TMPTA–PTSA–TMOS** cured films using **Cou-DCMC**-catalyzed sol–gel reactions.

4. ***Measurement Methods***

(a) An organic–inorganic solution was spin-coated on a polycarbonate substrate and heated at 60°C for a few minutes. The film was irradiated by 365 nm light under nitrogen gas. After that, all samples were covered with a fine layer (20 nm) of Au by sputtering and analyzed with SEM–EDX measurements. (i) Normalized line scan of silicon intensity in a cross-section of cured films from the upper to lower layer using SEM–EDX measurements. (ii) Elementary mapping images using SEM–EDX measurements.

(b) Organic or inorganic resins was spin-coated onto a substrate (polycarbonate or Si wafer). Contact angles measurements by applying 1.2 μL of ultrapure water, diiodomethane and ethylene glycol onto films. Surface free energy of all resins were measured using these contact angles.

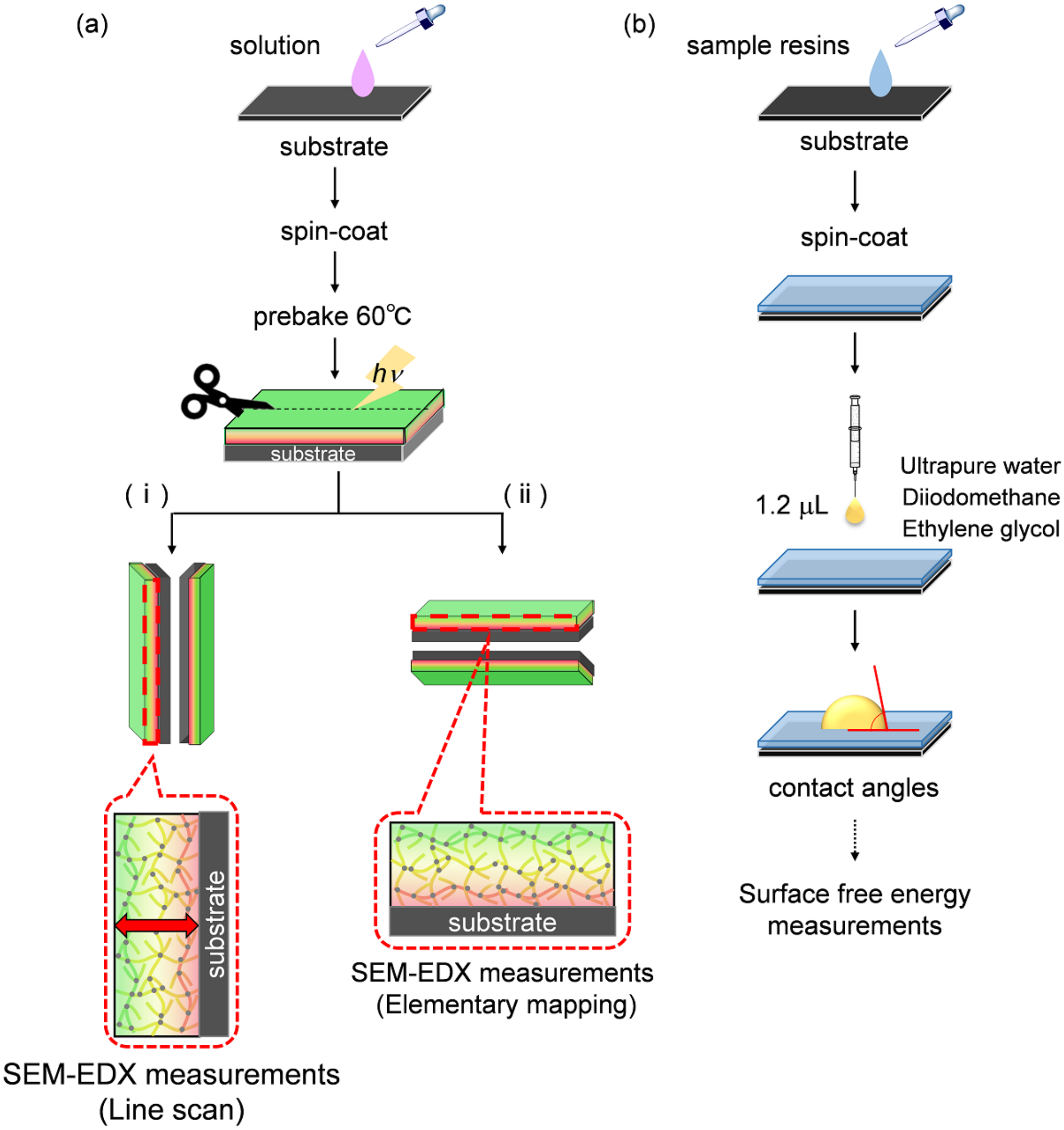

Figure S3. Measurement methods. (a) Line scan and elementary mapping measurement method using SEM–EDX measurements. (b) Surface free energy measurements method using contact angles.

5. Synthesis of Inorganic Oligomers

The inorganic oligomers were synthesized by using the sol–gel reaction.

5.1 PTSA

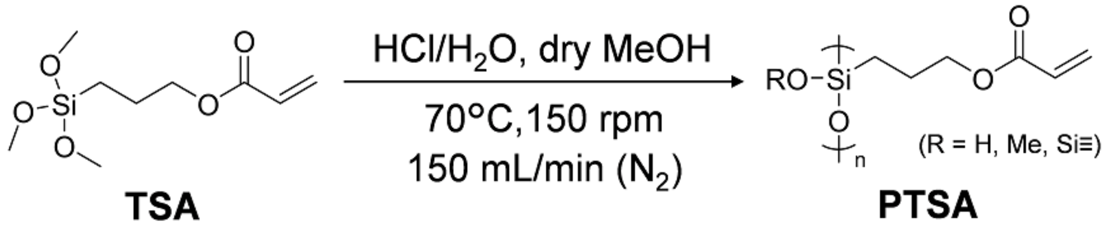

Super dehydrated methanol (40 mL) and **TSA** monomer (23.4 g, 100 mmol) were placed in a 300 mL three-necked flask equipped with a stirring rod, a nitrogen introduction tube, and an exhaust tube, and were then cooled with an ice bath for 10 min. Hydrochloric acid (1.09 g, 30 mmol) and ultrapure water 2.9 g (200 mmol) were added to this solution. After 10 min of stirring in an ice bath, stirring was continued for 10 min at room temperature. Hydrolytic polycondensation was then performed for 10 min via heating at 70°C in an oil bath at a stirring rate of 150 rpm and a nitrogen flow rate of 150 mL/min. The reaction mixture was dissolved in THF and removed from the three-necked flask. **PTSA** was reprecipitated in cyclohexane by condensation under reduced pressure and collected as a transparent, colorless, and viscous liquid. We obtained 16.5 g of **PTSA**. ^{1}H NMR (500 MHz, THF-d_8) 0.6–0.7 (br, 2H, *-CH$_3$-Si*), 1.7–1.8 (br, 2H, *-CH$_2$-CH$_2$-CH$_2$-*), 3.5–3.6 (br, 3H, *-O- [H or CH$_3$]*), 4.1–4.2 (br, 2H, *-CH$_2$-O-*), 5.82 (br, 1H, *-CH$_2$=CH-*), 6.1–6.2 (br, 1H, *=CH-*), 6.40 (br, 1H, *-CH$_2$=CH-*). The weight average molecular weight (M_w) and polydispersity index (M_w/M_n) were 9.5 × 10^3 and 2.2, respectively.

5.2 P(TSA–TFS)

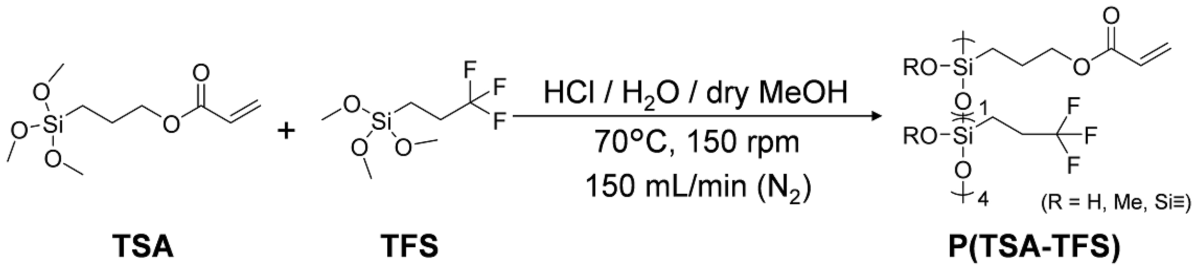

We repeated the procedure and molar ratio as per the **PTSA** synthesis, except that **TSA** monomer 4.68 g (20 mmol) and **TFS** monomer 17 g (80 mmol) was used instead of **TSA** monomer alone. Moreover, stirring was continued for 20 min at room temperature instead of 10 min, after 10 min of stirring in an ice bath. A total of 12.4 g of **P(TSA–TFS)** was obtained as a transparent, colorless, and viscous liquid. ^{1}H NMR (500 MHz, THF-d_8) 0.73 (br, 2H, *-CH$_2$-Si-*), 0.91 (br, 11H, *-CH$_2$-CF$_3$*), 1.8–1.9 (br, 2H, *-CH$_2$-CH$_2$-CH$_2$-*), 2.1–2.2 (br, 9H, *-Si-CH$_2$-CH$_2$-CF$_3$*), 3.5–3.8 (br, 8.7H, *-Si-O [H or CH$_3$]*), 4.15 (br, 2H, *-CH$_2$-O-*), 5.86 (br, 1H, *-CH$_2$=CH-*), 6.14 (br, 1H, *=CH*), 6.4–6.5 (br, 1H, *-CH$_2$=CH-*). The weight average molecular weight (M_w) and polydispersity index (M_w/M_n) were 6.5 × 10^3 and 1.3, respectively.

5.3 P(TSA–TFTS)

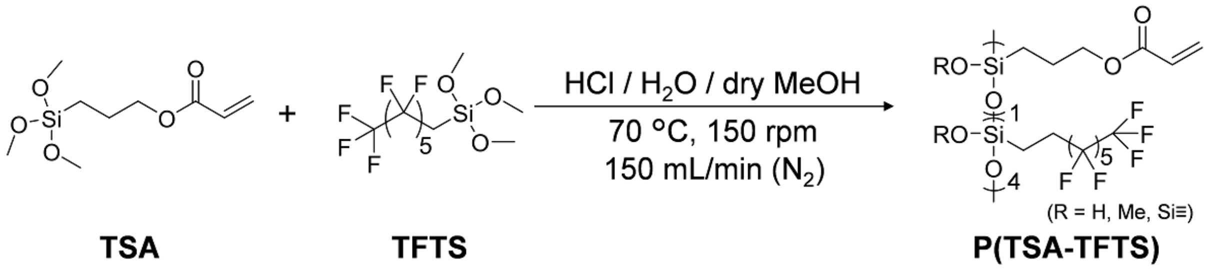

We repeated the procedure and molar ratio as per the **P(TSA–TFS)** synthesis, except that **TSA** monomer 0.94 g (4.0 mmol), and **TFTS** monomer 7.5 g (16 mmol) was used instead of **TFS** monomer. A total of 5.07 g of **P(TSA–TFS)** was obtained as a transparent, colorless, and viscous liquid. ^{1}H NMR (500 MHz, THF-d_8) 0.73 (br, 2.7H, *-CH₂-Si-*), 0.95 (br, 7.2H, *-CH₂-CF₃*), 1.8–1.9 (br, 2H, *-CH₂-CH₂-CH₂-*), 2.17 (br, 8H, *-Si-CH₂-CH₂-CF₂-*), 3.7–3.8 (br, 18H, *-Si-O [H or CH₃]*), 4.15 (br, 2H, *-CH₂-O-*), 5.86 (br, 1H, *-CH₂=CH-*), 6.14 (br, 1H, *=CH*), 6.4–6.5 (br, 1H, *-CH₂=CH-*). The weight average molecular weight (M_w) and polydispersity index (M_w/M_n) were 2.7×10^3 and 1.1, respectively.

5.4 P(TSA–PFPS)

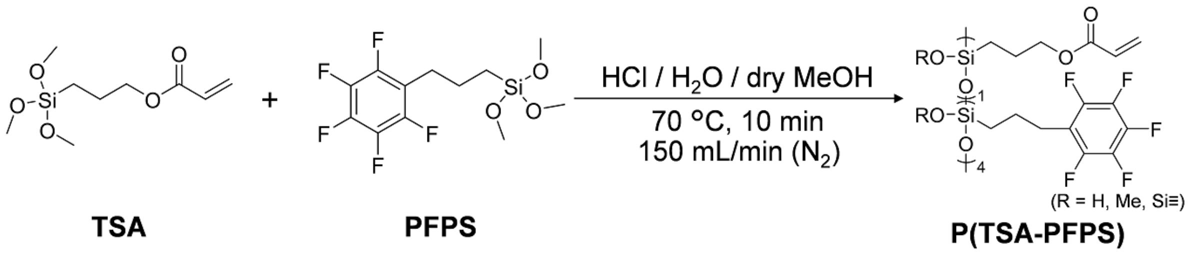

We repeated the procedure and molar ratio as per the **P(TSA–TFS)** synthesis, except that **TSA** monomer (8.4 mmol) and **PFPS** monomer (33 mmol) was used instead of **TFS** monomer. A total of 10.6 g of **P(TSA–PFPS)** was obtained as a transparent, colorless, and viscous liquid. Incidentally, **P(TSA–PFPS)** was not reprecipitated. ^{1}H NMR (500 MHz, THF-d_8) 0.6-0.7 (br, 12H, *-CH₂-Si-*, *-CH₂-Ar*), 1.8–1.9 (br, 16H, *-CH₂-CH₂-CH₂-*), 2.70 (br, 9.6H, *-Si-CH₂-CH₂-CH₂-*), 3.49 (br, 3.4H, *-Si-O [H or CH₃]*), 4.12 (br, 2.3H, *-CH₂-O-*), 5.84 (br, 1H, *-CH₂=CH-*), 6.11 (br, 1H, *=CH*), 6.3–6.4 (br, 1H, *-CH₂=CH-*). The weight average molecular weight (M_w) and polydispersity index (M_w/M_n) were 1.9×10^3 and 1.1, respectively.

6. *FT-IR Spectrometry for Radical Polymerization*

The spin-coated **TMPTA–PTSA** films on a silicon wafer were irradiated under nitrogen by 365 nm light of 0–15 J/cm^2. Figure S4 shows FT-IR spectra for a film consisting of **TMPTA** and **PTSA** after various doses of UV irradiation. As the film was irradiated by 365 nm light, the olefin peak (1650–1600 cm^{-1}) was decreased; therefore, radical polymerization progressed with olefin monomer consumption.

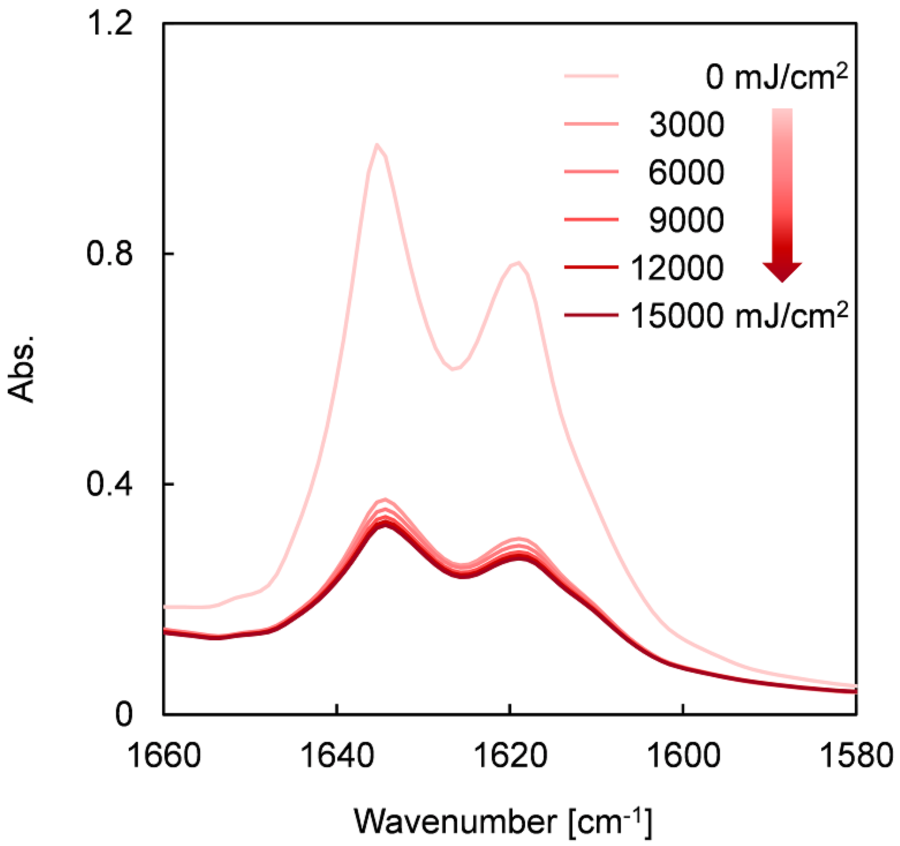

Figure S4. Effect of 365 nm light irradiation on FT-IR spectra for the spin-coated film consisting of **TMPTA** and **PTSA**. Abs. = absorbance.

7. ***Transparency of films***

The gradient and uniform dispersion films displayed colorless and transparent optical properties, respectively. However, the sea-island structured films, which are non-uniform are not transparent properties. The optical properties are one of the benefits that gradient structure provides.

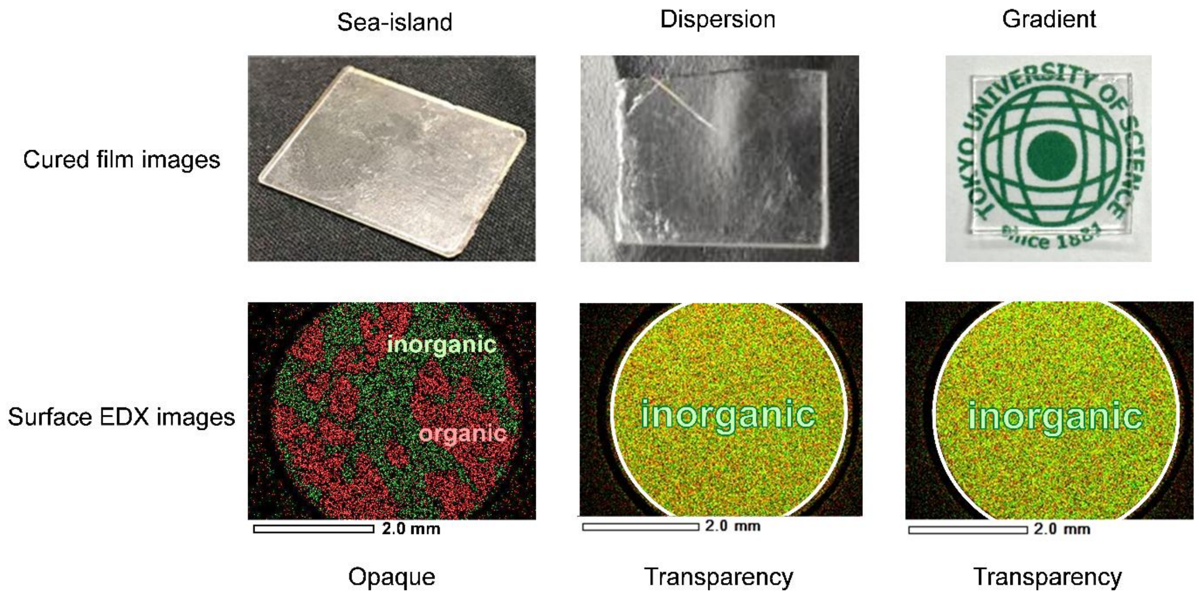

Figure S5. EDX images of the surface of the cured films (sea-island, dispersion, and gradient structure). EDX maps showing the distribution of silicon (Si), carbon (C).

8. *Dependence of Various Parameters for Cured Films*

8.1 Solvent effect

TMPTA (0.1 g) and **PTSA** (0.1 g) resin mixtures were dissolved in various solvents (minimum amount except CCl_4 0.1 g) containing Irgacure 819 (0.006 g). Cured films using these solutions were analyzed with SEM–EDX measurement in the same way as shown in Figure S3. All the resultant cured films displayed a gradient structure despite different solvent parameters, such as Rohrschneider's parameter and relative permittivity. This result indicates that the formation of the gradient structure does not depend on solvent properties, but removing solvent improves compatibility and viscosity change during heating.

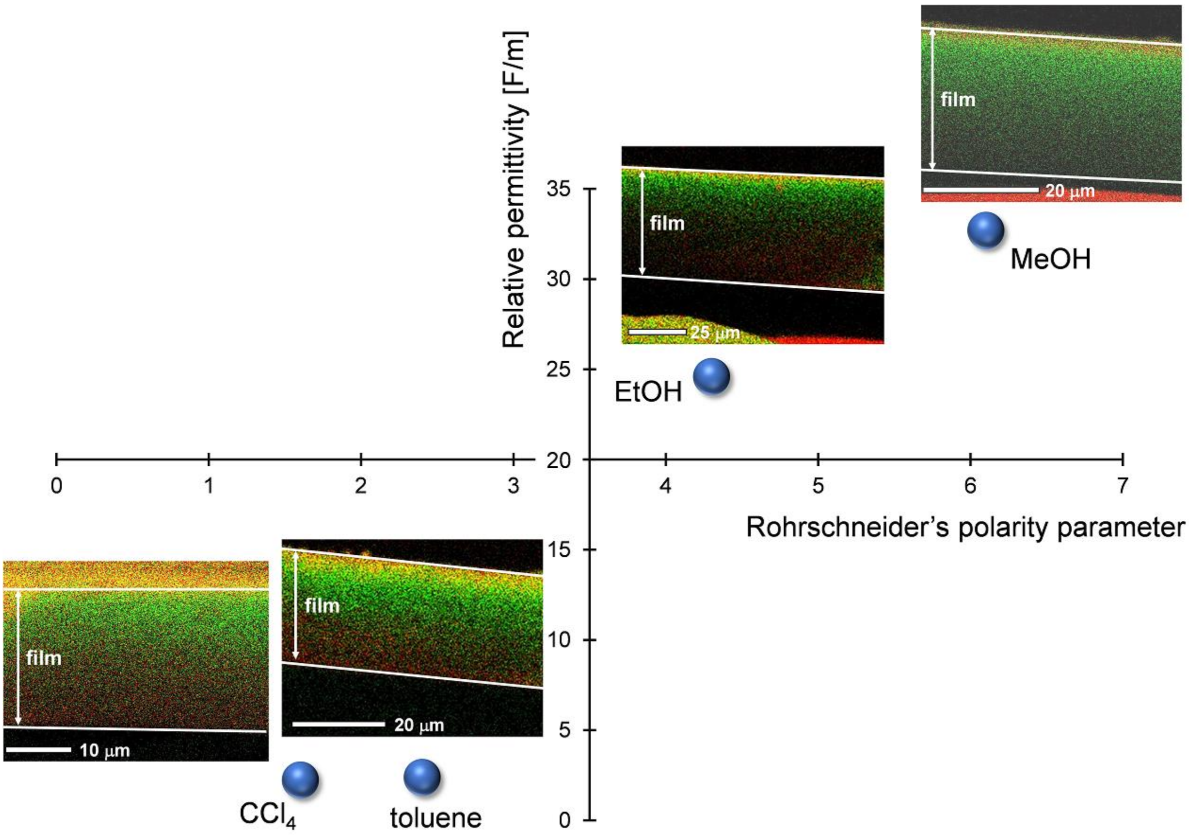

Figure S6. EDX images of the cross-section of the cured films using various solvents. EDX maps showing the distribution of silicon (Si), carbon (C).

8.2 Viscosity, surface free energy, and film thickness dependency of solvent-free films

TMPTA (0.1 g) containing Irgacure 651 (0.006 g) and **PTSA** (0.1 g) were mixed without solvent. The mixtures were spin-coated onto a polycarbonate substrate and heated at 60°C or stood at room temperature for 30 min. These films were irradiated under nitrogen by 365 nm light. These processes formed solventless films with various film thickness. Subsequently, all cured films were analyzed by SEM–EDX measurement.

Figure S7 shows correlation between viscosity ($\eta_{mix60°C}$: 67.7 mPa·s, $\eta_{mix25°C}$: 537 mPa·s) and film thickness (d:38-100 µm). High viscosity films formed dispersed structure in thick films, but gradient structure in thin films. This result is because thin films are affected by the surface free energy rather than viscosity, and thick films are affected by viscosity rather than surface free energy. Based on these results, thick films were heated at 60°C for 30 min because of the decreased viscosity of coating film. As a result, thick films formed the gradient structure, meaning that the film structure (dispersed or gradient) depend on surface free energy (film thickness) and viscosity either with or without the solvent.

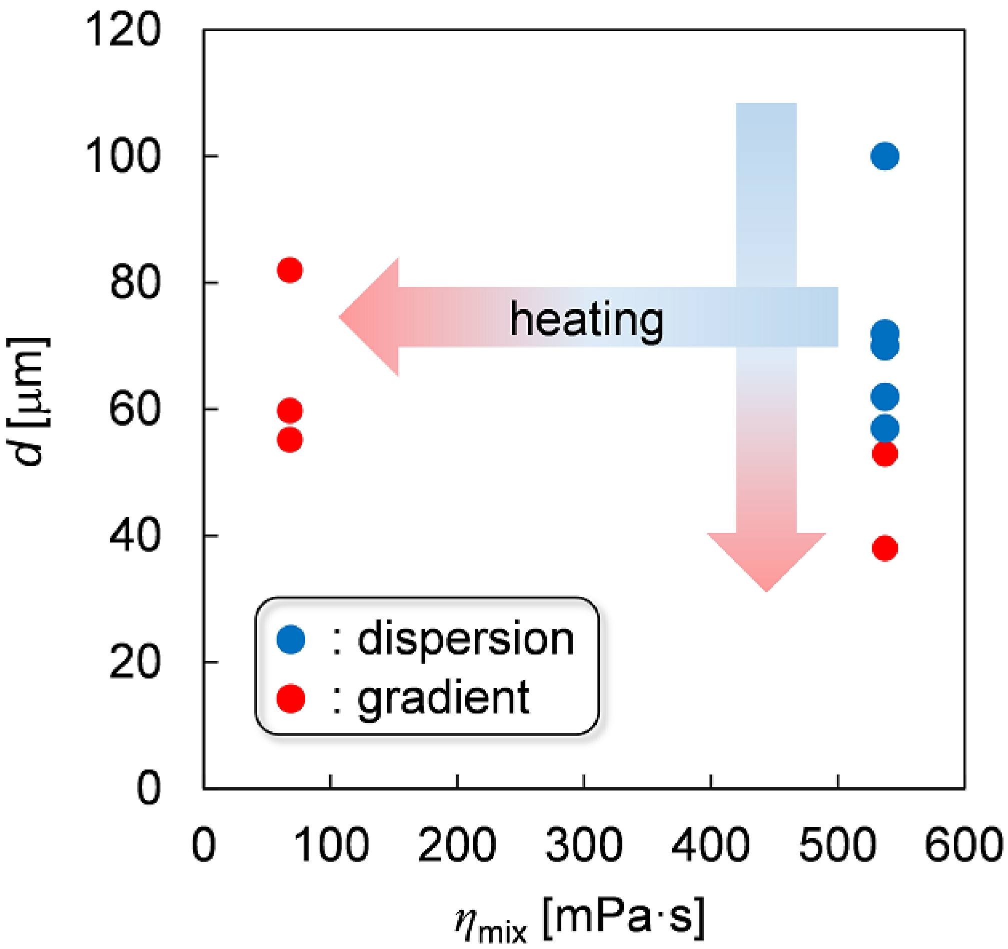

Figure S7. Correlation between viscosity and film thickness for **TMPTA–PTSA** films.

8.3 Standing time and film thickness dependency of solvent-free films

The same procedure as per the fabrication method of correlation between viscosity and film thickness was repeated, except that the coated films stood at room temperature for 0 or 30 min without heating.

Figure S8 shows a correlation between standing time (0 or 30 min) and film thickness (*d:* 18–100 µm) with the same viscosity (537 [mPa·s]).

All nonstanding films showed a dispersed structure by the effect of surface free energy because **PTSA** does not have enough time to affect surface free energy because of exposure immediately after film formation. By contrast, both thin and thick standing films displayed gradient and the dispersed structure, respectively. The formation of the dispersed structure is due to the surface free energy and viscosity. Specifically, suppression of resin movement by excessively high viscosity inhibited the effect of surface free energy in thick films. By contrast, thin films affected surface free energy implying an air interface effect compared with viscosity.

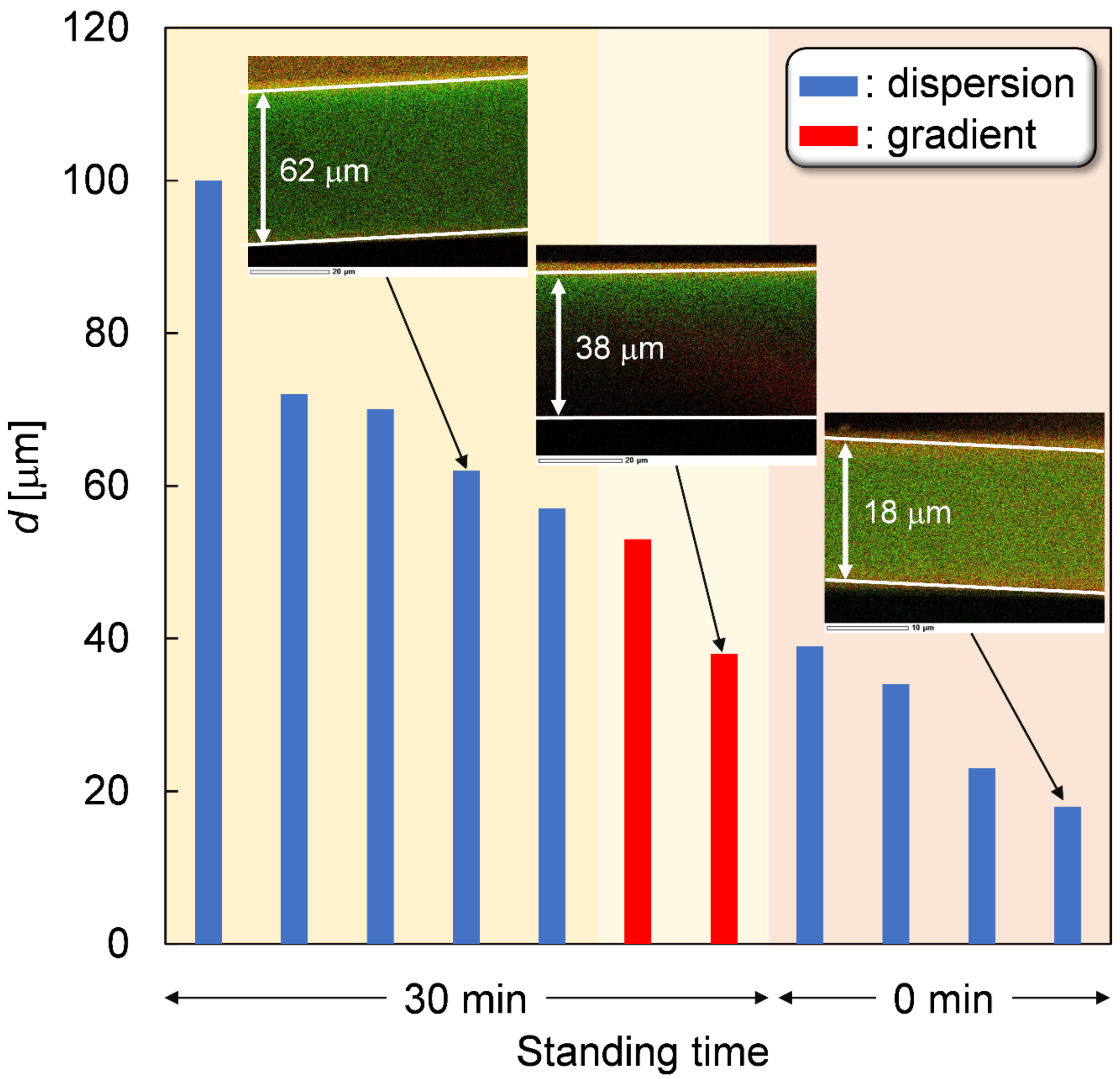

Figure S8. Correlation between standing time (surface free energy) and film thickness for **TMPTA–PTSA** films. EDX images of the cross-section of the cured films in each condition. EDX maps showing the distribution of silicon (Si), carbon (C).

8.4 Substrate dependency

The **TMPTA–PTSA** films spin-coated onto polycarbonate (PC) and polyethylene (PE) plates alone had a gradient structure, whereas the other substrates, namely acrylic plate, CaF_2 plate, glass plate, PE film, PET film, Si wafer, and PC plate, had dispersed structure (Figure S9). This result is because of interface free energy between resins and substrates.

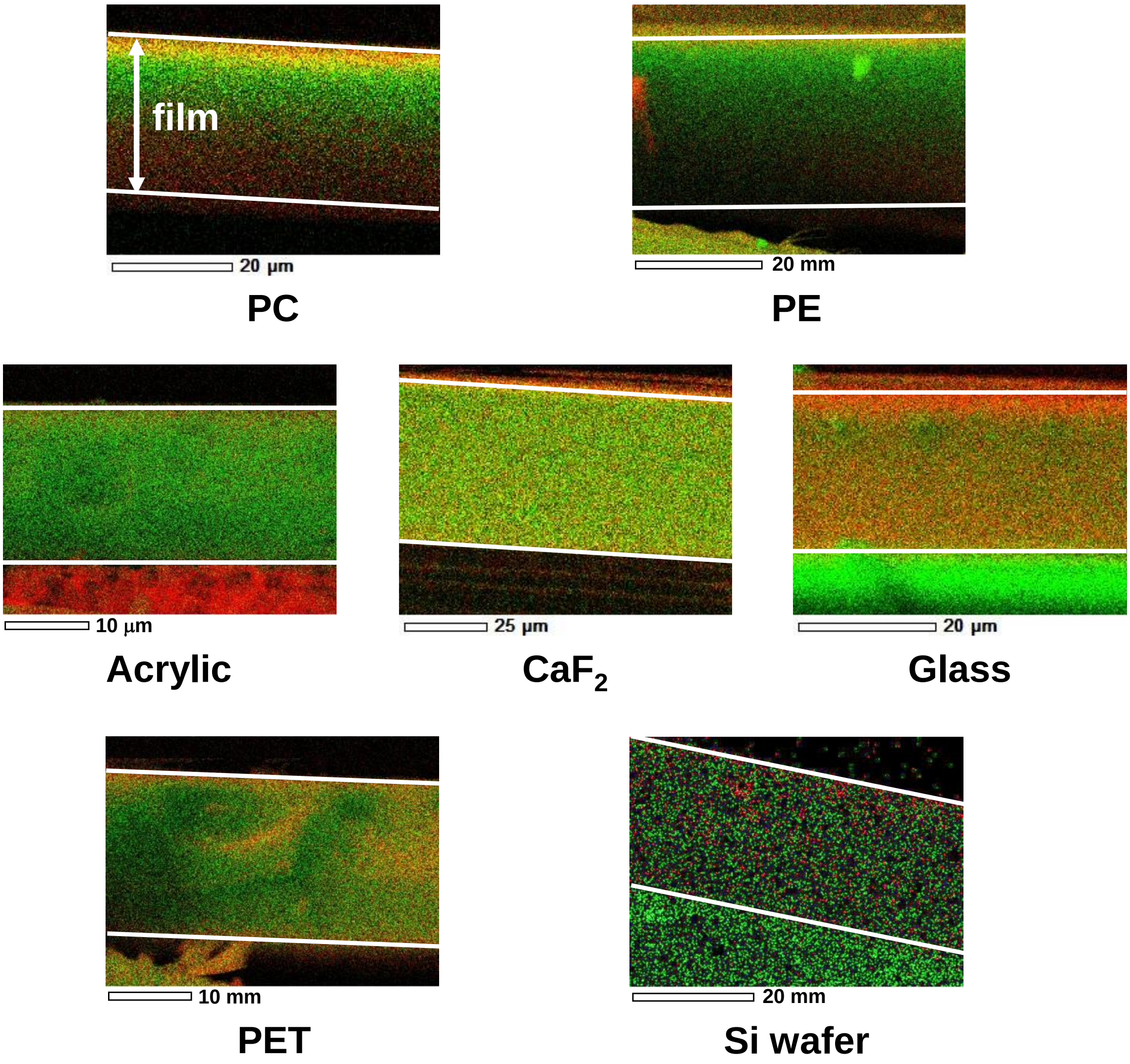

Figure S9. SEM–EDX images of cured **TMPTA–PTSA** films coated on various substrates.

8.5 Film thickness dependency on surface hardness and adhesion to organic substrate

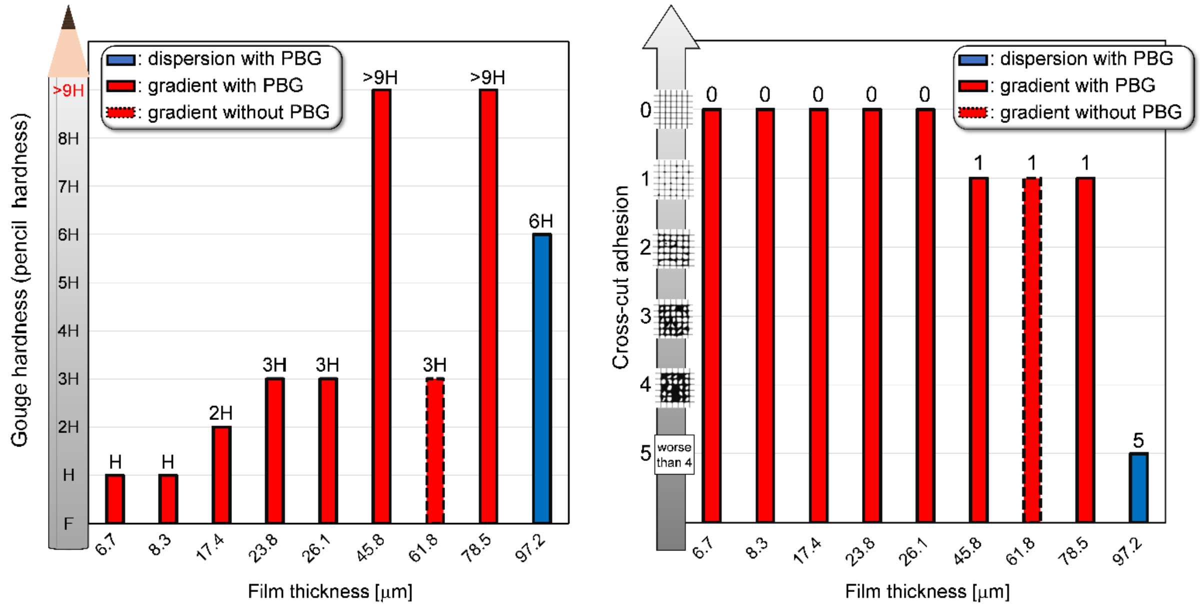

Figure S10. Pencil (gouge) hardness and cross-cut adhesion of the films in various conditions depending on the film thickness.

9. ***Various Parameters of All Organic and Inorganic Resins***

9.1 Viscosity and SP value of organic resins

Table S1. Various parameters of organic resins. Viscosity at 25ºC and 60°C, SP value by Fedors' method and molecular weight.

Organic resin	Viscosity [mPa·s]		SP value [$cal^{1/2}\cdot cm^{-3/2}$] (Fedors' method)[1]
	25°C	60°C	
**DPETHA**	6959	281.2	10.64
**DTMPTA**	987.0	80.34	10.09
**PETTA**	954.0	53.25	11.04
**PET3A**	852.8	45.01	11.72
**TMPTA**	106.1	14.52	10.47
**AHM**	49.65	8.738	11.70
**NGD**	9.017	3.777	9.770
**BenzA**	2.822	1.684	10.48

9.2 Viscosity and SP value of inorganic resins

Table S2. Various parameters of inorganic resins. Viscosity at 25°C and 60°C, SP value by Fedors' method and molecular weight.

Inorganic resin	Viscosity [mPa・s]		SP value [$cal^{1/2}\cdot cm^{-3/2}$] (Fedors' method)[2]	Weight average molecular weight (M_w)
	25°C	60°C		
**P(TSA–TFS)**	29300	1703	7.640	6.5×10^3
**PTSA**	4719	680	8.200	9.5×10^3
**P(TSA–TFTS)**	10130	600.4	7.530	2.7×10^3
**P(TSA–PFPS)**	4832	313.8	10.15	1.9×10^3
**MTPD (n = 130)**	153.9	80.7	7.450	1.0×10^4
**AHTPD**	164.5	47.17	9.809	600–900
**MTPD (n = 62)**	71.25	37.11	7.520	$4.5–5.5 \times 10^3$
**ATS**	13.33	6.235	7.754	570–620
**mMTPD**	5.37	3.14	7.796	600–800

9.3 Contact angles and surface free energy of organic resins

Table S3. Surface free energy of organic resins

Organic resin	Contact angle [°]			Surface free energy [mJ/m^2]	
	H_2O	Diiodomethane	Ethylene glycol	Acid–base[2]	Owens–Wendt[3]
**DPETHA**	50.2	21.8	—	—	57.4
**DTMPTA**	60.1	16.4	—	—	54.0
**PETTA**	48.1	10.7	—	—	60.1
**PET3A**	51.4	14.6	31.3	48.7	—
**TMPTA**	47.25	9.9	—	—	60.5
**AHM**	29.2	15.3	17.0	48.6	—
**NGD**	53.7	34.6	—	—	52.9
**BenzA**	90.7	22.5	—	—	47.3

9.4 Contact angles and surface free energy of inorganic resins

Table S4. Surface free energy of inorganic resins

Inorganic resin	Contact angle [°]			Surface free energy [mJ/m^2]	
	H_2O	Diiodomethane	Ethylene glycol	Acid–base[2]	Owens–Wendt[3]
**P(TSA–TFS)**	101.1	59.4	95.5	23.9	—
**PTSA**	57.7	40.3	44.5	39.4	—
**P(TSA–TFTS)**	112.4	95.0	88.0	11.4	—
**P(TSA–PFPS)**	70.1	48.5	46.7	38.4	—
**MTPD (n = 130)**	90.7	56.6	—	31.0	—
**AHTPD**	16.5	44.0	13.4	44.6	—
**MTPD (n = 62)**	78.4	55.4	—	34.6	—
**ATS**	86.3	41.3	—	—	39.2
**mMTPD**	77.8	41.9	—	—	40.4

10. Correlation Between Various Parameters and Film or Solution States

10.1 Correlation between solubility parameter difference, mixed viscosity at 60°C, and film states

Organic–inorganic films spin-coated onto a polycarbonate substrate analyzed using SEM–EDX. Figure S11 shows a scatterplot for correlation between solubility parameter difference, mixed viscosity at 60°C, and film states. Film states do not correlate with solubility parameter differences.

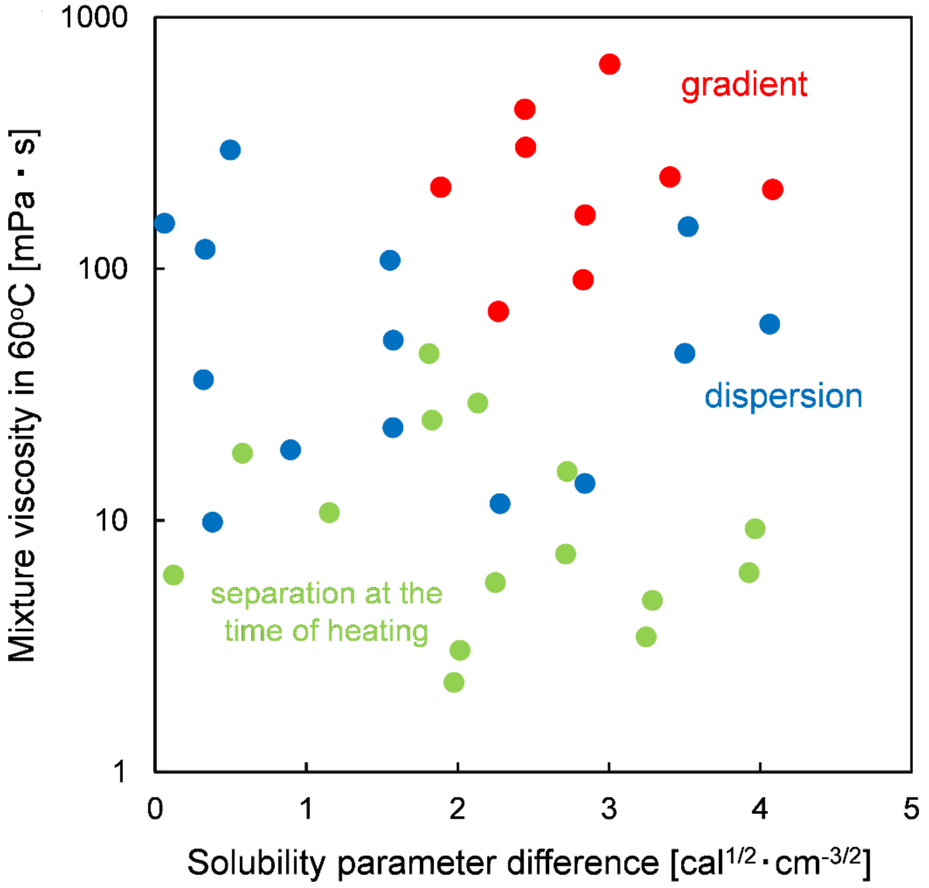

Figure S11. Scatterplots showing a lack of correlation between solubility parameter difference, mixture viscosity at 60°C, and membrane states (red, blue, and green plots represent gradient, dispersion, and separation, respectively).

10.2 Correlation between solubility parameter difference, mixed viscosity of organic resins, inorganic resins, and solvent (CCl_4) in 25°C and membrane states

Organic (0.1 g) and inorganic (0.1 g) resin mixtures were dissolved in carbon tetrachloride (0.2 g) containing Irgacure 819 (0.006 g). In this time, we observed the solution whether they are compatible or not. Figure S12 shows a scatterplot for correlation between solubility parameter difference, mixed viscosity of organic resins, inorganic resins, and solvent (CCl_4) at 25°C and membrane states. The compatibility of organic and inorganic resins depends on solubility parameter difference. When we use incompatible resin combinations, the solubility parameter difference is substantial. However, organic and inorganic resin combinations with a large solubility parameter difference are not necessarily incompatible.

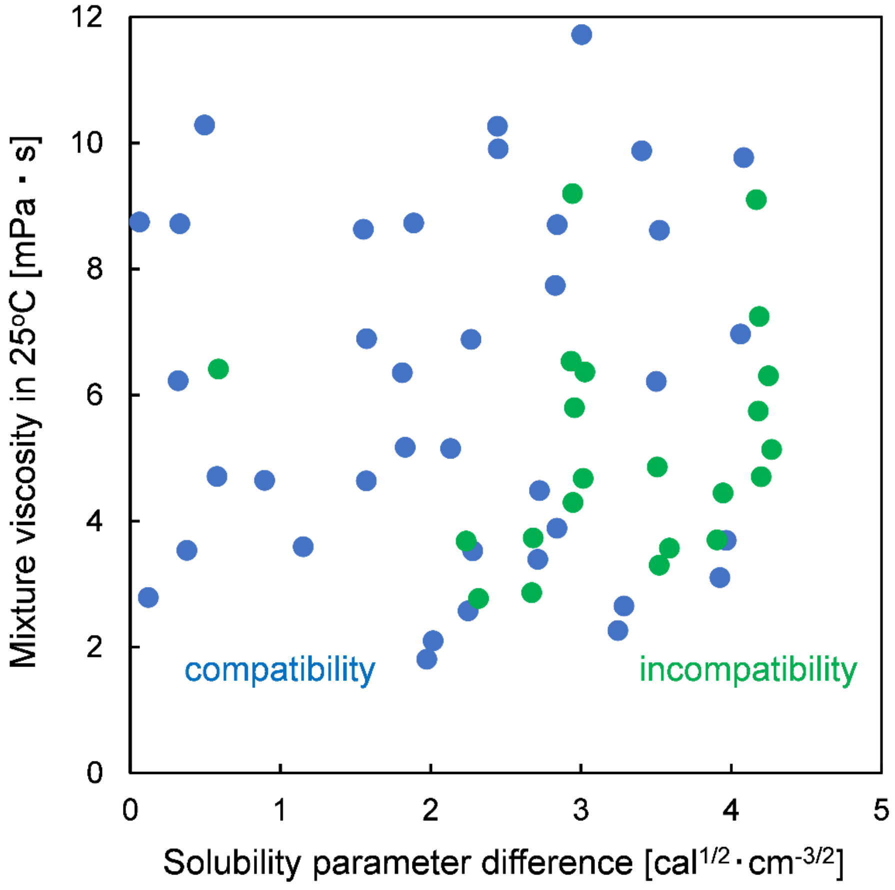

Figure S12. Scatterplot showing correlation between solubility parameter difference, mixed viscosity of organic resins, inorganic resins, and solvent (CCl_4) at 25°C, and film states (blue and green plots represent compatibility and incompatibility, respectively).

11. Mixed Viscosity

11.1 Mixed viscosity of organic resins and inorganic resins at 60°C

Table S5. Calculation of mixed viscosity of organic resins and inorganic resins at 60°C

Mixture viscosity [mPa·s]		Organic resin							
		**DPETHA**	**DTMPTA**	**PETTA**	**PET3A**	**TMPTA**	**AHM**	**NGD**	**BenzA**
Inorganic resin	**P(TSA–TFS)**	651	304	232	207	90.6	60.3	29.3	14.0
	**PTSA**	430	211	164	147	67.7	46.1	23.4	11.7
	**P(TSA–TFTS)**	—	—	156	140	65.0	44.4	22.7	11.3
	**P(TSA–PFPS)**	297	152	120	108	52.1	36.3	19.1	9.84
	**MTPD (n = 130)**	—	—	65.2	56.7	31.3	22.7	12.8	7.04
	**AHTPD**	—	—	50.1	46.1	25.0	18.5	10.7	6.07
	**MTPD (n = 62)**	—	—	44.3	40.8	22.6	16.8	9.87	5.66
	**ATS**	—	—	15.6	14.7	9.27	7.35	4.81	3.05
	**mMTPD**	—	—	9.83	9.30	6.20	5.04	3.44	2.27

11.2 Calculation method for organic and inorganic mixed viscosity

Organic and inorganic mixture viscosity was calculated using the following formula (S1.1)[4]:

$$\log(\log(\eta_{mix} + 1)) = \sum_{i=1}^{n} x_i(\log(\log(\eta_i + 1)))$$

Where each fraction of η_{mix}, η_i, x_i, and n indicate mixture viscosity, a viscosity of component i, a fraction of component i in the form of mass, and number of components in the mixture, respectively.

12. ***Film or Solution State for Each Mixed Resin***

All organic–inorganic solution was spin-coated on a polycarbonate substrate and heated at 60°C for 30 minutes. The film was irradiated by 365 nm light under nitrogen gas.

Table S6. Film or solution state for each mixed resin

Film or solution state		Organic resin							
		DPETHA	DTMPTA	PETTA	PET3A	TMPTA	AHM	NGD	BenzA
Inorganic resin	P(TSA–TFS)	G	G	G	G	G	D	S	D
	PTSA	G	G	G	D	G	D	D	D
	P(TSA–TFTS)	—	—	I	I	I	I	I	I
	P(TSA–PFPS)	D	D	D	D	D	D	D	D
	MTPD (n = 130)	—	—	I	I	I	I	I	I
	AHTPD	—	—	S	S	S	S	S	S
	MTPD (n = 62)	—	—	I	I	I	I	I	S
	ATS	—	—	S	I	S	S	S	S
	mMTPD	—	—	I	I	S	I	S	S

G, D, S, and I symbol show *G*radient, *D*ispersion, *S*eparation at the time of prebake and *I*ncompatibility, respectively.

Table S7. EDX images of the cross-section of the cured films. EDX maps showing the distribution of silicon (Si), carbon (C).

Film state		Organic resin		
		**P(TSA–TFS)**	**PTSA**	**P(TSA–PFPS)**
Inorganic resin	**DPETHA**	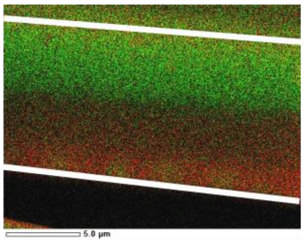 Gradient	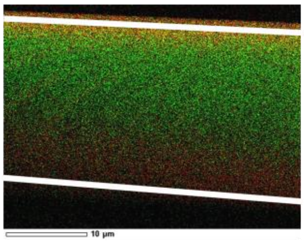 Gradient	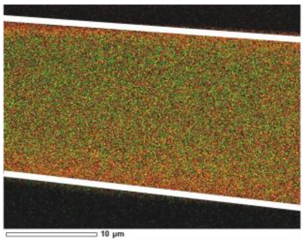 Dispersion
	**DTMPTA**	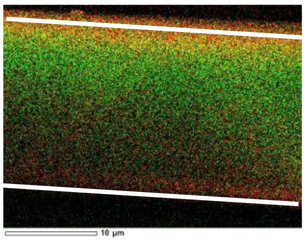 Gradient	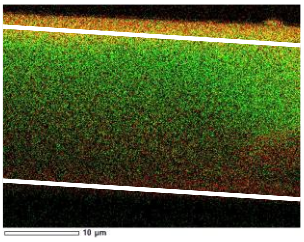 Gradient	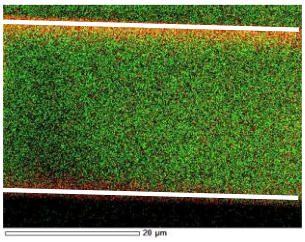 Dispersion
	**PETTA**	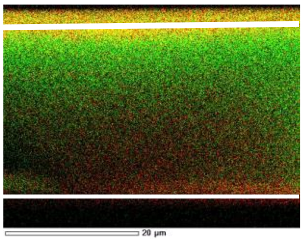 Gradient	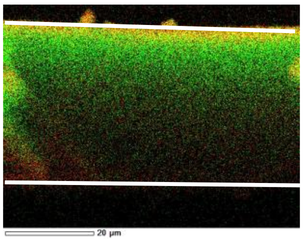 Gradient	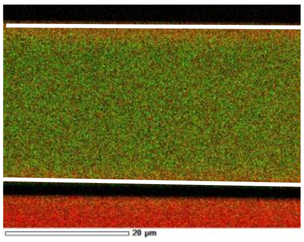 Dispersion
	**PET3A**	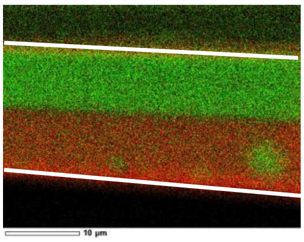 Gradient	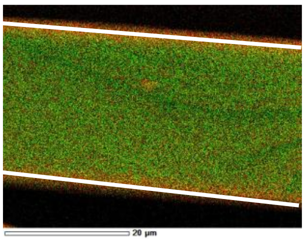 Dispersion	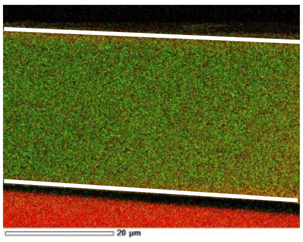 Dispersion

Table S7. (Continued)

Film state		Organic resin		
		**P(TSA–TFS)**	**PTSA**	**P(TSA–PFPS)**
Inorganic resin	**TMPTA**	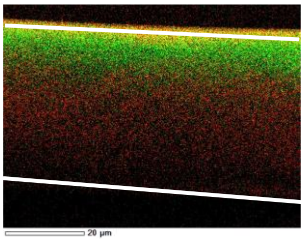 Gradient	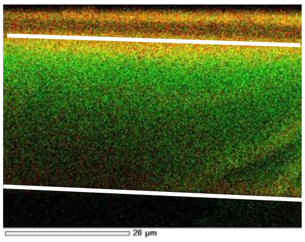 Gradient	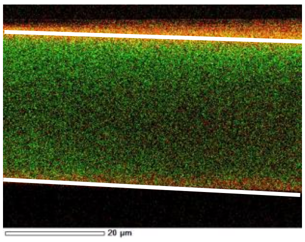 Dispersion
	**AHM**	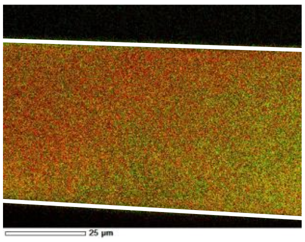 Dispersion	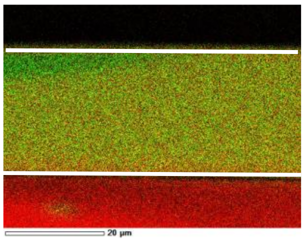 Dispersion	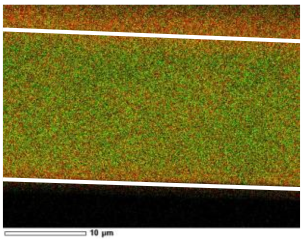 Dispersion
	**NGD**	Separation	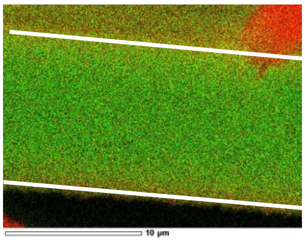 Dispersion	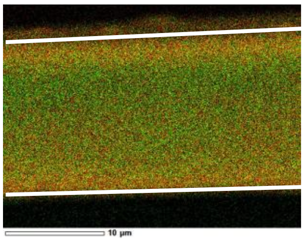 Dispersion
	**BenzA**	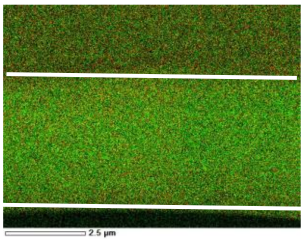 Dispersion	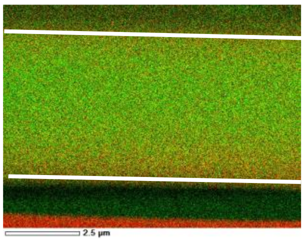 Dispersion	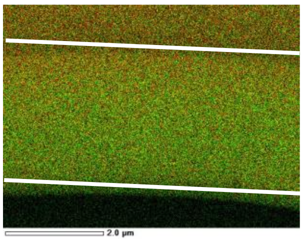 Dispersion

13. ***Pencil hardness test***

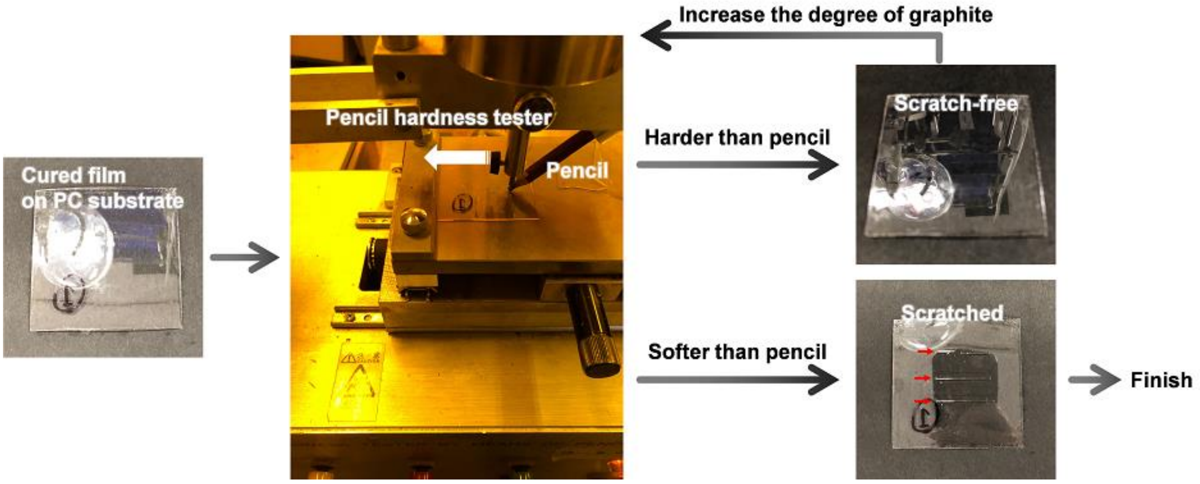

Figure S13. Photographs of pencil hardness test. Red arrows guided scratches of a cured film.

14. Abbreviations and Structures of the Chemical Compounds

Table S8. Abbreviations and structures of the chemical compounds used in this work.

Chemical name	Abbreviation	Chemical structure
1-(Acryloyloxy)-3-(methacryloyloxy)-2-propanol	AHM	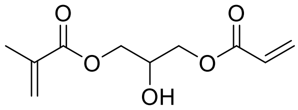
1,9-Bis(acryloyloxy)nonane	NGD	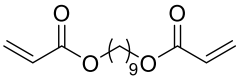
2-Acetoxycinnamic acid	2Aca-acid	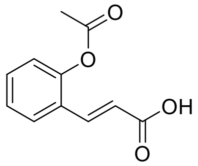
2,2-Dimethoxy-1,2-diphenylethan-1-one	Irgacure 651	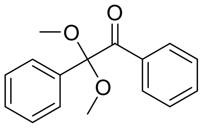
(3-acryloxy-2-hydroxypropoxypropyl) terminated polydimethylsiloxane	AHTPD	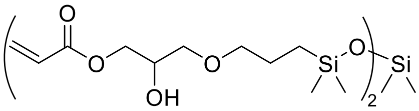
3-(Trimethoxysilyl)propyl acrylate	TSA	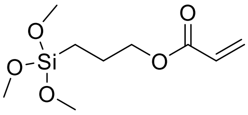
Acryloxypropyl t-structure siloxane	ATS	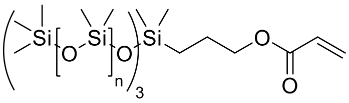
Benzyl acrylate	BenzA	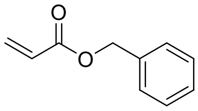
Bis(2,4,6-trimethylbenzoyl) phenylphosphine oxide	Irgacure 819	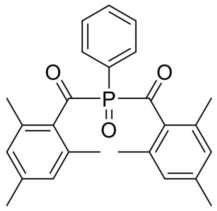
Dipentaerythritol hexaacrylate	DPETHA	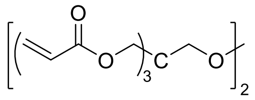
Ditrimethylolpropane tetraacrylate	DTMPTA	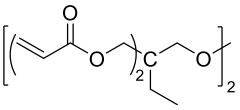
Methacryloxypropyl terminated polydimethylsiloxane	MTPD	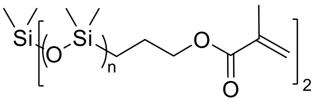
Monomethacryloxypropyl terminated polydimethylsiloxane	mMTPD	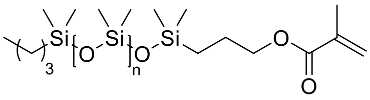

N,*N′*-Dicyclohexyl-4-morpholinecarboxamidine	DCMC	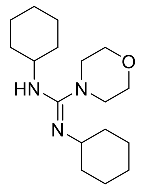
Pentaerythritol tetraacrylate	PETTA	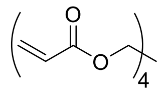
Pentaerythritol triacrylate	PET3A	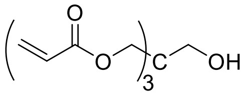
Pentafluorophenylpropyltrimethoxysilane	PFPS	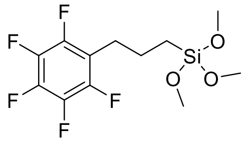
Trimethoxy(1*H*,1*H*,2*H*,2*H*-tridecafluoro-*n*-octyl) silane	TFTS	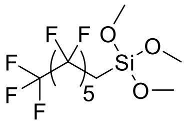
Trimethoxy(3,3,3-trifluoropropyl) silane	TFS	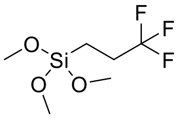
Trimethylolpropane triacrylate	TMPTA	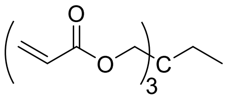
Coumaric-*N*,*N′*-dicyclohexyl-4-morpholinecarboxamidine	Cou-DCMC	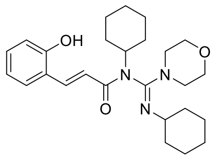
Poly(3-(trimethoxysilyl)propyl acrylate)	PTSA	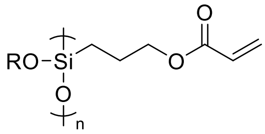
Poly(3-(trimethoxysilyl)propyl acrylate-pentafluorophenylpropyltrimethoxysilane)	P(TSA–PFPS)	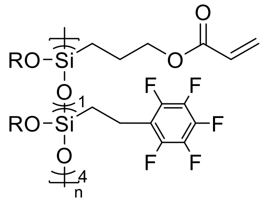
Poly(3-(trimethoxysilyl) propyl acrylate-trimethoxy(1*H*,1*H*,2*H*,2*H*-tridecafluoro-1-octyl) silane)	P(TSA–TFTS)	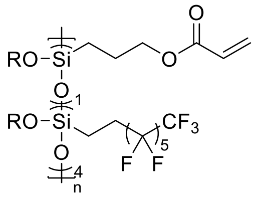
Poly(3-(trimethoxysilyl) propyl acrylate-trimethoxy(3,3,3-trifluoropropyl) silane)	P(TSA–TFS)	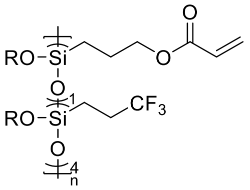

15. References

1. Fedors, R. F. A method for estimating both the solubility parameters and molar volumes of liquids. *Polym. Eng. Sci.* **1974**, *14*, 147–154.
2. Lee, L. H.; Mittal, K. L. *Contact Angle, Wettability and Adhesion*; Utrecht, **1993**, p. 45.
3. Owens, D. K.; Wendt, R. C. Estimation of the surface free energy of polymers. *J. Appl. Polym. Sci.* **1969**, *13*, 1741–1747.
4. Zhang, L.; Xu, Z.; Guo, X.; Xu; C.; Zhao; S. Viscosity mixing rule and viscosity–temperature relationship estimation for oil sand bitumen vacuum residue and fractions. *Energy Fuels* **2018**, *33*, 206–214.
